# Loneliness as a predictor of posttraumatic stress disorder symptom change among treatment-seeking US military veterans

**DOI:** 10.1017/S0033291726105157

**Published:** 2026-07-22

**Authors:** Lauren Sippel, Reanne Cunningham Chilton, Amy Hoang, David Rozek, Noelle Smith, Ilan Harpaz-Rotem, Rani Hoff

**Affiliations:** 1 Geisel School of Medicine at Dartmouth, USA; 2 VA Connecticut Healthcare System, USA; 3 VA San Diego Healthcare System, USA; 4 University of Texas at San Antonio Health Science Center, USA; 5 VA Northeast Program Evaluation Center, USA; 6 Yale School of Medicine, USA

**Keywords:** dysphoric arousal, loneliness, longitudinal, PTSD, veterans

## Abstract

**Background:**

Among military veterans, loneliness is common and associated with poorer mental and physical health and functioning. Epidemiological studies have revealed bidirectional, longitudinal associations between loneliness and posttraumatic stress disorder (PTSD). Little is known about loneliness among treatment-seeking veterans, how loneliness impacts PTSD symptom change during treatment, and whether loneliness has differential associations with specific PTSD symptom clusters.

**Method:**

Data were derived from the Veterans Outcome Assessment, an annual, repeated-measures (baseline and 3-month follow-up) telephone survey of patients beginning new episodes of mental health care in the Department of Veterans Affairs. Participants were 1,155 veterans (23% women) entering PTSD specialty care. We tested whether baseline loneliness predicted change in total PTSD symptom severity and according to the five-factor Dysphoric Arousal model of PTSD using hierarchical regressions in which we statistically adjusted for demographics and mental health encounters.

**Results:**

Loneliness decreased between baseline and 3-month follow-up (*d* = 0.39). Baseline loneliness predicted change in PTSD for the dysphoric arousal and negative alterations in cognitions and mood symptom clusters, with lonelier veterans demonstrating more PTSD change between baseline and follow-up. However, lonelier veterans also had more severe PTSD symptoms at both timepoints. This pattern of results held even after accounting for different types of treatment encounters between timepoints.

**Conclusions:**

While lonelier veterans have more severe PTSD symptoms, they also make greater improvements in symptoms salient to interpersonal functioning over time. Assessment of loneliness should be incorporated into PTSD treatment planning and considered as a treatment target that could be addressed to promote recovery.

## Introduction

Loneliness has been defined as distress due to the discrepancy between desired and perceived interpersonal relationships (Cacioppo & Cacioppo, 2018). It is related to, but distinct from, objective social isolation, which corresponds to the number of people in an individual’s environment. Because loneliness reflects perceived disconnection rather than objective contact, it may persist even when individuals are surrounded by others and may be especially clinically relevant for individuals with mental health conditions like posttraumatic stress disorder (PTSD), where avoidance, mistrust, and emotional numbing can disrupt the capacity to feel socially connected. In 2023, loneliness was identified by the US Surgeon General as an ‘epidemic’ that requires attention and intervention (United States Department of Health and Human Services, 2023). This conclusion was based on research showing that loneliness is associated with a host of negative physical and mental health outcomes, including but not limited to increased all-cause mortality (Holt-Lunstad et al., 2010), cardiovascular disease (Teshale et al., 2023), dementia (Kuiper et al., 2015), depression (Mann et al., 2022), and risk of suicide (Teo et al., 2018), as well as fewer prosocial behaviors and lower social self-efficacy (Palmer et al., 2020), and poorer academic and work performance (Bowers et al., 2022; Guay et al., 1999).

### Loneliness among veterans

Loneliness and these associated negative outcomes are highly prevalent among military veterans (Wilson et al., 2018). Findings from a nationally representative sample reveal that nearly 40% of veterans report moderate to high levels of loneliness (Ong et al., 2024), with severity of loneliness associated with increased odds of difficulties with mental health, physical health, and functioning (Schafer et al., 2024). Also among veterans, being married/partnered and perceiving greater purpose in life were associated with reduced loneliness over time, whereas poorer cognitive functioning, psychological distress (including symptoms of PTSD), and having experienced more childhood adversity predicted greater increases in loneliness (Ong et al., 2024). Veterans who reported feeling lonely ‘sometimes’ and ‘often’ were 3 and 12 times more likely to endorse suicidal ideation, respectively, compared to veterans who reported ‘hardly ever’ feeling lonely (Straus et al., 2022).

### PTSD and loneliness

As noted, one of the mental health conditions associated with loneliness is PTSD (Solomon et al., 2014), which is highly prevalent among veterans (Lehavot et al., 2018). PTSD is characterized by intrusions of traumatic memories, avoidance of trauma reminders, negative thoughts and feelings, and hyperarousal – all of which could serve to isolate veterans from others. Among those experiencing PTSD symptoms, loneliness may also increase the risk for other difficulties, such as suicidal ideation (Levi-Belz et al., 2024).

Changes in PTSD symptoms over time are associated with changes in loneliness over time (Fox et al., 2021). For example, loneliness predicts subsequent increases in PTSD symptoms (Boyraz, 2026). There is little evidence that increases in PTSD predict increases in loneliness, though a similar association has been observed in studies of social support (Wang et al., 2021). PTSD may increase loneliness via trauma-related social withdrawal, and loneliness may confer risk for, or maintain, PTSD through negative cognitions about the self and others. The cognitive theory of PTSD (Ehlers & Clark, 2000) purports that negative appraisals of trauma sequelae lead to a sense of ‘current threat’ that drives the onset and maintenance of PTSD. Negative posttrauma cognitions such as ‘People don’t want to be around me’ may serve to promote social isolation even as one may desire engagement in relationships – thereby leading to loneliness. Similarly, loneliness and social exclusion (whether real or perceived) can be experienced as interpersonal threats and heighten hypervigilance to negative social information (Langenkamp et al., 2025), which could then interfere with engagement in relationships and even treatment.

PTSD has highly heterogeneous presentations due to the many combinations of symptoms that can lead to a diagnosis of PTSD. Assessing the potentially distinct relationships between PTSD symptom clusters and loneliness could inform more tailored treatment planning. For example, loneliness may be most closely tied to symptom domains that directly shape interpersonal engagement (e.g. detachment, restricted affect, and irritability) rather than to fear-based symptoms alone, which would have implications for case conceptualization and adjunctive targets.

The *DSM-5* includes four PTSD symptom clusters: intrusions, avoidance, negative alterations in cognition and mood, and alterations in arousal and reactivity (American Psychiatric Association, 2013). Some studies suggest that alternative factor structures (ranging from 2 to 8) may better capture PTSD heterogeneity than the *DSM-5* four-cluster organization (Rasmussen et al., 2019). To our knowledge, only one study has examined whether the association between loneliness and PTSD symptoms varies by PTSD symptom cluster. The authors found that, in a convenience sample of veterans who had served in Iraq or Afghanistan, only the negative alterations in mood and cognitions (NACM) symptom cluster was associated with greater loneliness, both cross-sectionally and one year later (Lwi et al., 2023).

### Loneliness and PTSD treatment

PTSD is a highly treatable condition, with evidence supporting both psychological and pharmacological interventions. However, little is known about the extent to which loneliness intersects with PTSD treatment outcomes – either in the general population or among veterans who have ready access to PTSD treatment through the Department of Veterans Affairs (VA). Studying loneliness in the context of treatment initiation is particularly informative because it allows evaluation of whether loneliness changes alongside early symptom improvement and whether baseline loneliness signals a different symptom trajectory during PTSD care. Such knowledge could inform screening and assessment efforts and could help providers anticipate challenges and/or opportunities during treatment planning.

A recent cross-sectional analysis of treatment-seeking veterans in the United Kingdom, the majority of whom reported PTSD symptoms, revealed that almost 80% of veterans experienced loneliness, which was associated with poorer mental health (Williamson et al., 2023). To our knowledge, no studies to date have tested whether loneliness improves during treatment focused on PTSD in usual care settings.

### The current study

Little is known about loneliness among veterans seeking PTSD treatment, how loneliness may impact PTSD symptom change during mental health care, and whether loneliness has differential associations with specific PTSD symptom clusters. Using data collected from veterans entering a new episode of care in VA outpatient specialty PTSD services, we seek to extend previous work on PTSD and loneliness by examining these associations among treatment-seeking veterans using a longitudinal design. This work is intended to inform the degree of need for incorporating loneliness into treatment planning and intervention. We hypothesized the following: (1) loneliness would improve from baseline to 3-month follow-up, and (2) greater loneliness at baseline would be associated with less improvement in overall PTSD symptom severity (and especially negative cognition and mood and hyperarousal symptoms) even when adjusting for demographic characteristics and overall utilization of mental health care.

## Method

### Participants and procedures

Study data were procured from the Veterans Outcome Assessment (VOA), an annual, repeated measures (baseline and 3-month follow-up) telephone survey of patients beginning a new episode of VA mental health care (Katz et al., 2020). The VOA was first approved in 2017 by the US Office of Management and Budget to collect interview data on 10,000, later increased to 15,000, veterans annually (for VOA methods, see Katz et al., 2020, 2021, 2022). The VOA routinely collects data on veterans entering the following programs for the purpose of program evaluation: General Mental Health, Primary Care-Mental Health Integration, outpatient Specialized PTSD, Specialized Substance Use Disorder, and veterans discharged from acute inpatient units.

The current study reports on veterans entering outpatient Specialized PTSD services in VOA wave 7 (which crossed fiscal years 2023–2024). Eligibility criteria for being selected to be contacted for a VOA interview were: (1) no participation in specialized PTSD treatment in the past six months, and (2) at least two current outpatient PTSD visits (to exclude those seen solely for PTSD assessment). Thus, in this context, a new episode of care reflects initiation of specialty PTSD treatment following at least six months without prior PTSD specialty services, rather than first lifetime treatment. Eligible patients were identified weekly, based on automatic screening of the electronic medical record, and veterans were contacted by external contracting staff within 2 weeks. Interviews lasted approximately 30 minutes, and trained interviewers identified themselves as VA representatives and used a computer-assisted protocol. Veterans were informed that participation was voluntary, no compensation would be given, and that any data collected would not be available to their clinical providers (except for instances of clinical risk management). Patients unable to be contacted within 30 days of the eligibility visit were excluded.

The study was approved and deemed exempt from informed consent by the VA Connecticut Healthcare System IRB.

### Measures

Demographic and clinical data, including sex, age, race, ethnicity, marital status, employment status, rurality, psychiatric diagnoses, and mental healthcare utilization (i.e. encounters), were extracted from the electronic health record. Combat history was derived from the VOA interview data.

#### PCL-5

The PTSD Checklist for *DSM-5* (PCL-5; Blevins et al., 2015), a 20-item measure assessing the 20 symptoms of PTSD was administered at baseline and follow-up. The PCL-5 is widely used for monitoring PTSD symptom change during and after treatment, and has well-established psychometric properties (Bovin et al., 2016; National Center for PTSD, 2025). Likert scale items are summed to produce a total score ranging from 0 to 80; internal consistency in the current study was excellent (baseline PCL-5 *α* = .93, follow-up PCL-5 *α* = .96). We used confirmatory factor analysis (CFA) to identify the best-fitting measurement model of PTSD symptom clusters for this sample. We chose CFA because the PCL-5 has been subjected to extensive study within similar populations to our own, making this theory-driven approach appropriate. We prioritized testing models that have been supported in studies of treatment-seeking veterans (e.g. the five-factor Dysphoric Arousal model; Sippel et al., 2019) rather than models that were based on nationally representative samples (e.g. the seven-factor model; Armour et al., 2016) or that are more novel (the eight-factor model; Gross et al., 2023), in part because these models with a greater number of factors, each composed of a few items, may have limited clinical significance (Rasmussen et al., 2019) – making them a poorer fit for our objectives.

#### Campaign to end loneliness measurement tool

The Campaign to End Loneliness Measurement Tool (Campaign to End Loneliness, 2019), a 3-item scale developed to measure change in the context of interventions to address loneliness was given at baseline and follow-up. This tool measures loneliness indirectly insofar as respondents rate how strongly they agree or disagree with items that do not include the word ‘lonely’ and are positively valenced. Likert items ranging from ‘strongly agree’ (0) to ‘strongly disagree’ (4) are summed to produce a total score ranging from 0 to 12, with higher scores indicative of greater loneliness. Internal consistency in the current study was acceptable to good (baseline *α* = .77, follow-up *α* = .82). The Campaign to End Loneliness Tool was selected by VA to assess loneliness in the VOA because of its brevity and appropriateness for older adults, who are over-represented in the veteran population. This scale does not have validated cut scores for determining the presence/absence of loneliness.

### Analyses

All analyses were conducted using SAS. The sample was described using a combination of self-reported data and data derived from medical records. Sample description variables included race/ethnicity, sex (male/female), age category (at baseline interview), marital status (married or unmarried at the time of the baseline interview), employment status (employed or unemployed at the time of the baseline interview), rurality (dichotomized as living in a rural or nonrural area at the time of the baseline interview), and combat history (dichotomized as having experienced combat or not). Sample description variables reported reflect the full baseline sample (1155 and involved minimal missing data). No imputations were completed for missing data. Of the 1,155 baseline participants, 722 (62.5%) had a matched follow-up. Attrition analyses indicated that participants with and without follow-up did not differ significantly on gender, race/ethnicity, marital status, rurality, inpatient status, baseline loneliness, baseline PTSD symptom severity, PTSD symptom clusters, or encounter variables (all *p*’s > .05). However, follow-up completion differed by age category, χ^2^(7) = 16.26, *p* = .023, and combat exposure, χ^2^(1) = 5.82, *p* = .016, such that older participants and those with combat exposure were somewhat more likely to complete follow-up. Effect sizes for these associations were small (Cramer’s Vs = .12 and .07, respectively). The number of encounters coded as being related to mental health that occurred between baseline and follow-up was included as an additional covariate. To qualify as a mental health encounter, the associated encounter code had to pertain to either an individual mental health therapy, group therapy, or psychiatric medication-related appointment. An overall encounter variable, as well as each of the above three encounter type variables, was used for analyses. Encounter variables were winsorized to reduce the effect of extreme values.

We first conducted CFA to identify the best-fitting measurement model of PTSD symptom clusters on which to complete the rest of our planned analyses. We next conducted dependent-sample t-tests to compare baseline and follow-up loneliness and PCL-5 scores (total and factor-level). We then conducted six hierarchical regressions examining how baseline loneliness influenced PCL-5 raw difference change scores, adjusting for the number of mental health encounters and patient variables (age, sex, race/ethnicity, marital status, rurality, and combat history). All covariates were entered into the model in the same step. Six hierarchical regression analyses were performed using all identified covariates in the first step and those covariates, in addition to baseline loneliness, in the second step to predict change in the PCL-5 total and in each of the factors of the PCL-5 established with the CFA. Notably, the PCL-5 change scores were inverted such that a higher/more positive change score reflected more improvement/greater symptom reduction.

To aid interpretation, we also divided baseline loneliness into tertiles (low, medium, and high baseline loneliness). Analyses of variance (ANOVAs) were used to compare loneliness tertiles on baseline and follow-up PCL-5 scores.

Finally, we examined the effect of encounter type (individual therapy, group therapy, and medication appointment) on outcomes by rerunning our hierarchical regressions, replacing the total encounters variable with each of these variables in separate models.

## Results

### Description of sample

Sample descriptives are presented in Table 1. The baseline sample included 1155 veterans, and follow-up included 722 veterans. Veterans were predominantly White, male, combat-exposed, between the ages of 36–65 and living in an urban environment. At baseline, veterans reported moderately severe PTSD symptoms (*M* = 44.95, *SD* = 16.72) and moderate levels of loneliness (*M =* 5.47, *SD* = 2.91). Between baseline and follow-up, 65% of the sample had at least one psychotherapy encounter at the VA.

**Table 1.** Sample characteristics Table 1. long description.

Baseline characteristics	Frequency (percentage adjusted for missing *n*’s *^a^* ^–^ *^h^*)
Sex	1,150 *^a^*
Male	883 (76.8%)
Female	267 (23.2%)
Race and ethnicity	1,155 *^b^*
White	590 (51.1%)
American Indian and Alaska Native	12 (1.0%)
Asian American	19 (1.7%)
Black/African American	269 (23.3%)
Native Hawaiian and Pacific Islander	12 (1.0%)
Hispanic/Latinx	100 (8.7%)
More than one race	116 (10.0%)
Unknown	37 (3.2%)
Age	1,148 *^c^*
18–25	35 (3.1%)
26–35	171 (14.9%)
36–45	289 (25.2%)
46–55	227 (19.8%)
56–65	241 (21.0%)
66–75	128 (11.2%)
76+	57 (5.0%)
Marital status	1,121*^d^*
Married	576 (51.4%)
Not married	545 (48.6%)
Employment	1,153 *^e^*
Employed	552 (47.9%)
Not employed	601 (52.1%)
Rurality	1,112 *^f^*
Urban	839 (75.5%)
Rural+	273 (24.6%)
Combat history	1,155 *^g^*
Yes	746 (64.6%)
No	409 (35.4%)
**Encounters**	
Total Mental Health, winsorized	1145 *^h^*
Mean (SD)	9.88 (9.93)
1 or more encounters	1022 (88.48%)
>2 encounters	973 (84.24%)
Individual psychotherapy, winsorized	1145 *^h^*
1 or more encounters	835 (72.29%)
>2 encounters	754 (65.28%)
Group psychotherapy, winsorized	1145 *^h^*
1 or more encounters	263 (22.77%)
>2 encounters	229 (19.83%)
Medication Management, winsorized	1145 *^h^*
1 or more encounters	578 (50.04%)
>2 encounters	439 (38.01%)

*Note: N* = 1,155 veterans (full sample). Percentages for each category are based on the total number of responses (excluding those missing *^a–h^*). *^f^* ‘Rural’ included any veteran whose area code fell within the VA Office of Rural Health (https://www.ruralhealth.va.gov/aboutus/ruralvets.asp) and Planning System Support Group’s definitions of ‘rural’, ‘highly rural’, or ‘insular island’. *^g^* Encounters indicates the encounters that occurred between baseline and follow-up. Any encounters in psychotherapy included individual therapy, group therapy, and medication management visits but did not include assessment, case management, nor primary care visits which may have addressed mental health.

a Missing values for race and ethnicity: n = 0.

b Sex: n = 5.

c Age: n = 7.

d Marital status: n = 34.

e Employment: n = 2.

f Rurality: n = 43.

g Combat history: n = 0

h Psychotherapy: n = 10.

### Measurement model

The five-factor Dysphoric Arousal model (for *DSM-IV*: Elhai et al., 2011; for *DSM-5:* Demirchyan et al., 2015) had a slightly better fit (χ^2^ = 851.193, df = 160, CFI = 0.9344, TLI = 0.9221, RMSEA = 0.0627) than the four-factor Emotional Numbing model of PTSD symptom structure (for DSM*-IV*: King et al., 1998; for *DSM-5*: Demirchyan et al., 2015) (χ^2^ = 828.8754, df = 129, CFI = 0.9267, TLI = 0.9131, RMSEA = 0.0702) and the two-factor Information Processing model (Horowitz, 1979) (χ^2^ = 1306.3368, df = 169, CFI = 0.892, TLI = 0.8786, RMSEA = 0.0783) as indicated by the CFI, TLI, and RMSEA values. At follow-up, the five-factor structure remained slightly stronger (χ^2^ = 668.8005, df = 160, CFI = 0.9497, TLI = 0.9403, RMSEA = 0.0678) than the four-factor structure (χ^2^ = 621.1198, df = 129, CFI = 0.9459, TLI = 0.9358, RMSEA = 0.0742) and the two-factor structure (χ^2^ = 955.6634, df = 169, CFI = 0.9222, TLI = 0.9126, RMSEA = 0.0821).

For these reasons, we conducted all PTSD factor-level analyses using the five factors from the Dysphoric Arousal model: Reexperiencing (intrusive thoughts, nightmares, flashbacks, emotional cue reactivity, and physiological cue reactivity); Avoidance (avoidance of thoughts and reminders); Negative alterations to cognitions and mood (NACM; trauma-related amnesia, negative beliefs, blame of self or others, negative trauma-related emotions, loss of interest, detachment, and restricted affect); Anxious arousal (hypervigilance and exaggerated startle response); and Dysphoric arousal (irritability/anger, self-destructive/reckless behavior, difficulty concentrating, and sleep disturbance). Additionally, this model was selected to balance empirical fit with clinical interpretability of symptom clusters relevant to interpersonal functioning.

### Associations between loneliness and PTSD symptoms

As shown in Table 2, loneliness and PTSD symptom severity were positively and significantly correlated at both timepoints. T-tests for differences in loneliness and PCL-5 scores at baseline and follow-up are presented in Table 3. There was a statistically significant, small-to-medium-sized reduction in loneliness from baseline to follow-up magnitude (*d* = 0.39). PTSD symptom severity decreased by almost 9 points from baseline to follow-up (*d* = 0.47). There were statistically significant reductions in all five PTSD symptom clusters (*d* range = 0.34–0.44).

**Table 2.** Bivariate correlations between loneliness and PTSD symptoms at baseline and three-month follow-up Table 2. long description.

Timepoint and measure	*M* (*SD*)	Correlations												
		1.	2.	3.	4.	5.	6.	7.	8.	9.	10.	11.	12.	13.
Baseline														
1. Loneliness	5.47 (2.91)	1.00												
2. Total PCL-5	44.95 (16.72)	.44^**^	1.00											
3. Reexperiencing	2.29 (1.01)	.33^**^	.86^**^	1.00										
4. Avoidance	2.65 (1.17)	.29^**^	.75^**^	.63^**^	1.00									
5. NACM	2.15 (0.97)	.49^**^	.91^**^	.65^**^	.59^**^	1.00								
6. Anxious	2.68 (1.14)	.22^**^	.72^**^	.58^**^	.49^**^	.55^**^	1.00							
7. Dysphoric	1.98 (0.88)	.38^**^	.82^**^	.60^**^	.50^**^	.70^**^	.55^**^	1.00						
Follow-up														
8. Loneliness	4.32 (2.92)	.43^**^	.30^**^	.22^**^	.17^**^	.32^**^	.14*	.22^**^	1.00					
9. Total PCL-5	36.17 (20.73)	.28^**^	.48^**^	.40^**^	.33^**^	.42^**^	.35^**^	.41^**^	.54^**^	1.00				
10. Reexperiencing	1.92 (1.17)	.21^**^	.44^**^	.43^**^	.32^**^	.35^**^	.34^**^	.35^**^	.43^**^	.91^**^	1.00			
11. Avoidance	2.15 (1.35)	.22^**^	.36^**^	.32^**^	.30^**^	.31^**^	.27^**^	.29^**^	.46^**^	.85^**^	.77^**^	1.00		
12. NACM	1.71 (1.12)	.34^**^	.47^**^	.35^**^	.29^**^	.47^**^	.29^**^	.39^**^	.57^**^	.94^**^	.78^**^	.75^**^	1.00	
13. Anxious	2.15 (1.32)	.14*	.37^**^	.31^**^	.26^**^	.27^**^	.42^**^	.28^**^	.35^**^	.83^**^	.71^**^	.66^**^	.72^**^	1.00
14. Dysphoric	1.56 (1.01)	.25^**^	.44^**^	.34^**^	.28^**^	.38^**^	.29^**^	.45^**^	.49^**^	.89^**^	.74^**^	.71^**^	.81^**^	.71^**^

*Note:* Loneliness = loneliness score from *The Campaign to End Loneliness Tool.* PCL-5 = PTSD total and symptom-based factor scores from the PTSD Checklist for *DSM-5* (Blevins et al., 2015). A five-factor model based on discrete symptom clusters (Reexperiencing, Avoidance, Negative Alterations to Cognitions and Mood [NACM], Anxious Arousal, and Dysphoric Arousal) was fitted to data using confirmatory factor analysis.

^*^p < .001. ^**^p < .0001.

**Table 3.** Dependent samples t-tests comparing baseline versus follow-up loneliness and PCL-5 scores Table 3. long description.

Measure	Baseline				Follow-up				*t*	Effect size, *d*
	*M*	*SD*	Range	*n*	*M*	*SD*	Range	*n*		
Loneliness	5.47	2.91	0–12	1,146	4.32	2.92	0–12	714	9.65^**^	.3945
PCL-5 factors										
Reexperiencing	2.29	1.01	0–4	1,142	1.92	1.17	0–4	714	9.09^**^	.3411
Avoidance	2.65	1.17	0–4	1,140	2.15	1.35	0–4	712	9.20^**^	.3981
NACM	2.15	0.97	0–4	1,141	1.71	1.12	0–4	711	9.98^**^	.4159
Anxious	2.68	1.14	0–4	1,140	2.15	1.32	0–4	710	10.91^**^	.4267
Dysphoric	1.98	0.88	0–4	1,140	1.56	1.01	0–4	710	10.74^**^	.4422
PCL-5 total scores	44.95	16.72	0–80	1,099	36.17	20.73	0–80	692	11.97^**^	.4658

*Note*: Loneliness = loneliness score from *The Campaign to End Loneliness* Tool. PCL-5 = PTSD total and symptom-based factor scores from the PTSD Checklist for *DSM-5* (Blevins et al., 2015). A five-factor model based on discrete symptom clusters (Reexperiencing, Avoidance, Negative Alterations to Cognitions and Mood [NACM]; Anxious Arousal, Dysphoric Arousal) was fitted to data using confirmatory factor analysis.

** *p* < .0001.

### Examination of loneliness as a predictor of PTSD symptom change

**Figure 1.**
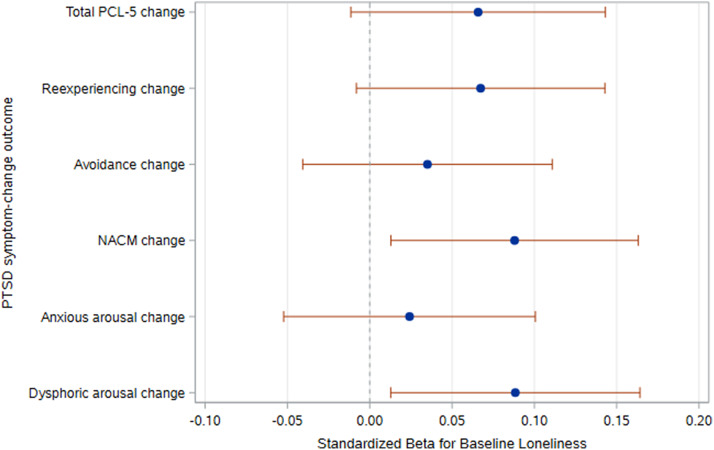
Standardized associations between baseline loneliness and symptom improvement. *Note:* *p < .05. Figure 1. long description.

Hierarchical regression analyses examining how loneliness predicted PTSD symptom change are presented in Table 4. Analyses for the change in each of the five symptom clusters are presented in Supplementary Tables A–E. Baseline loneliness significantly predicted PCL-5 change scores above and beyond the effect of the covariates for NACM (*p* = .0220) and Dysphoric Arousal (*p* = .0234) symptom clusters, but not total PCL-5 (*p* = .0956), Reexperiencing (*p* = .0808), Avoidance (*p* = .3647), or Anxious Arousal (*p* = .4416). The positive associations between baseline loneliness and change in NACM and dysphoric arousal symptom severity are displayed in Figure 1.

**Table 4.** Hierarchical regression predicting total PCL-5 score change, based on loneliness, demographics covariates at baseline, and total encounters Table 4. long description.

Predictor	Step 1 (covariates only)				Step 2 (covariates plus loneliness)			
	*β*	SE	*t*	*p*	*β*	SE	*t*	*p*
Intercept	17.50	8.78	1.99	.0468	14.94	8.91	1.68	.0941
Age group								
26–35	−5.27	4.39	−1.20	.2305	−5.12	4.39	−1.16	.2445
36–45	−1.23	4.24	−.29	.7715	−1.32	4.24	−.31	.7556
46–55	−4.73	4.27	−1.11	.2685	−4.76	4.27	−1.11	.2655
56–65	−6.40	4.20	−1.52	.1286	−6.30	4.21	−1.50	.1350
66–75	−8.95	4.49	−1.99	.0468	−8.74	4.49	−1.94	.0523
76+	−11.43	5.13	−2.23	.0262	−11.14	5.14	−2.17	.0304
Race and ethnicity								
American Indian and Alaska Native	7.85	7.91	.99	.3212	7.62	7.91	.96	.3359
Asian American	−.996	5.08	−.20	.8447	−.239	5.26	−.05	.9637
Black/African American	3.40	1.95	1.75	.0811	3.14	1.95	1.60	.1091
Native Hawaiian and Pacific Islander	−6.43	7.30	−.88	.3792	−6.82	7.31	−.93	.3513
Hispanic/Latinx	−4.25	2.86	−1.49	.1373	−4.04	2.86	−1.41	.1587
Other race/ethnicity	3.40	4.26	.80	.4245	3.54	4.26	.83	.4068
Unknown	−.735	2.60	−.28	.7778	−.781	2.62	−.30	.7654
Marital status	1.24	1.56	.79	.4285	1.58	1.58	1.00	.3187
Sex	3.68	1.86	1.97	.0490	3.65	1.87	1.95	.0514
Rurality	3.92	1.79	2.18	.0294	4.01	1.80	2.23	.0262
Combat history	1.81	1.59	1.14	.2566	1.62	1.60	1.01	.3117
Total encounters	.081	.071	1.14	.2536	.070	.071	.98	.3251
Loneliness, baseline	–	–	–	–	.423	.253	1.67	.0956
Model R^2^				.052				.056

*Note:* Positive β values indicate greater PTSD symptom improvement (i.e. larger reduction in PCL-5 scores).

Reference categories: age (18–25), race/ethnicity (white), married, male, urban setting, and no combat history.

**Figure 2.**
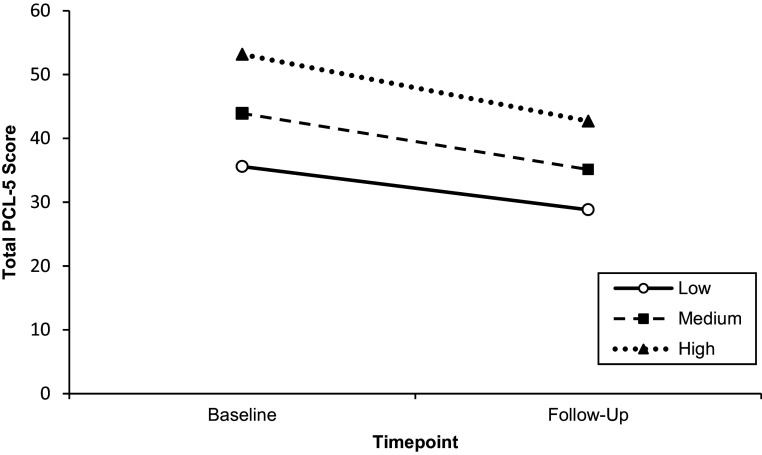
Mean total PTSD scores by loneliness tertiles (low, medium, and high). *Note*: Mean total scores on the *PTSD Checklist for the DSM-5* (PCL-5; Blevins et al., 2015) across low, medium, and high tertiles of loneliness are plotted against study timepoint (baseline and follow-up). Figure 2. long description.

To further probe this finding, as shown in Table 5 and Figure 2, ANOVAs comparing the three loneliness tertiles (low, medium, and high baseline loneliness) revealed that lonelier veterans had higher PCL-5 scores (total and all five factors, as shown in Supplementary Figures A–E) both at baseline and follow-up. Tukey’s HSD post hoc procedure revealed that all loneliness tertiles were significantly different across all variables at baseline and follow-up, with the exception of follow-up Anxious Arousal; the medium loneliness group was not different from the high loneliness group, though it did differ from the low loneliness group.

**Table 5.** ANOVAs examining PCL-5 scores by loneliness tertiles Table 5. long description.

Dependent variable	DF	Sum of squares (Model)	Mean square (Model)	F Value	Pr > F	Error mean square	DF error	Corrected total
Baseline PCL-5 total	2	52095.95	26047.98	111.74	<.0001	233.12	1091	306428.53
Follow-up PCL-5 total	2	22662.11	11331.05	28.48	<.0001	397.92	684	294840.51
Baseline reexperiencing	2	116.74	58.37	64	<.0001	0.91	1130	1147.33
Follow-up reexperiencing	2	43.44	21.72	16.45	<.0001	1.32	706	975.43
Baseline avoidance	2	109.91	54.96	43.45	<.0001	1.26	1129	1537.96
Follow-up avoidance	2	65.23	32.62	18.79	<.0001	1.74	704	1287.14
Baseline NACM	2	220.51	110.26	145.15	<.0001	0.76	1130	1078.85
Follow-up NACM	2	91.69	45.85	40.63	<.0001	1.13	703	885.02
Baseline anxious arousal	2	59.38	29.69	23.59	<.0001	1.26	1129	1480.41
Follow-up anxious arousal*	2	27.15	13.58	7.97	0.0004	1.70	702	1222.53
Baseline dysphoric arousal	2	109.53	54.77	80.65	<.0001	0.68	1129	876.16
Follow-up dysphoric arousal	2	43.15	21.57	22.64	<.0001	0.95	702	711.99

*Note:* *Tukey’s HSD post hoc procedure revealed that all loneliness tertile groups differed significantly across all variables at baseline and follow-up, with the exception of Follow-up Anxious Arousal; the Medium loneliness group was not different from the High loneliness group, though did differ from Low loneliness.

### Regression models by MH encounter type

To test whether the findings depended on the type of mental health care veterans received, we reran the hierarchical regressions, replacing the overall encounter count with each encounter type (individual psychotherapy, group psychotherapy, and medication management) in separate models (results are presented in Supplementary Tables F–W). Across all three modalities, baseline loneliness remained a significant predictor of greater improvement in the NACM and dysphoric arousal symptoms and did not significantly predict change in total PTSD severity or the other symptom clusters. Thus, the association between baseline loneliness and symptom change was not attributable to any single type of treatment contact.

## Discussion

In this first longitudinal study of PTSD symptoms and loneliness among treatment-seeking veterans, we found that more lonely veterans were more symptomatic than less lonely veterans at baseline and follow-up and that loneliness modestly decreased over time. These findings extend both cross-sectional (Solomon et al., 2014; Straus et al., 2022) and longitudinal work (Ong et al., 2024) showing that PTSD symptoms are associated with greater loneliness among veterans by demonstrating that this relationship persists over time among veterans who are starting mental health treatment for PTSD.

We also found that lonelier veterans showed greater improvement in symptoms in the dysphoric arousal and NACM symptom clusters above and beyond the effect of the total number of mental health encounters and the type of mental health encounters (individual therapy, group therapy, or medication management), meaning that social contact with providers during utilization of mental health care was not the only driver of this effect. This finding is partially consistent with those of Lwi et al., 2023, who found that only the NACM symptom cluster was associated with greater loneliness among veterans. Importantly, baseline severity and symptom change are distinct: lonelier veterans were more symptomatic at both timepoints, consistent with our hypothesis that loneliness marks poorer overall status, yet showed greater change specifically in the NACM and dysphoric arousal clusters. This pattern is plausible given that these clusters carry the highest interpersonal loading and may be especially responsive to the social contact inherent in initiating mental health treatment. The loneliness association concentrated in precisely these interpersonally salient domains, rather than across all symptoms, suggests a targeted relationship between loneliness and the PTSD symptoms most tied to social connection. Previous work has shown that emotional numbing (which is composed of symptoms in the *DSM-5* NACM cluster) is most strongly associated with poor intimacy, and hyperarousal symptoms (many of which are reflected in the Dysphoric Arousal cluster) are most closely linked to perpetration of intimate partner violence (Birkley et al., 2016). Similarly, previous work in veterans in residential treatment for PTSD has shown unique associations between emotional numbing and dysphoric arousal symptoms and poorer social connectedness (Sippel et al., 2019). Taken together, these findings suggest that trauma-related negative beliefs and emotions may indeed be bidirectionally associated with poorer social connectedness among veterans and serve as a meaningful and modifiable treatment target.

### Limitations and future directions

While our longitudinal design is a strength of the current study, no causal inferences can be made since this study did not have an experimental manipulation. Broader inclusion criteria and a relatively short assessment period (i.e. veterans in any current outpatient PTSD treatment examined at baseline and 3-month follow-up) allowed us to capture a larger, more clinically diverse sample of veterans who could have received PTSD treatment of any nature from any VA. Notably, the association between baseline loneliness and change in negative alterations in cognitions and mood and dysphoric arousal symptoms held when the overall encounter count was replaced by individual psychotherapy, group psychotherapy, or medication management encounters in separate models, indicating that this finding was not driven by any single type of treatment contact. Nonetheless, encounter counts remain a coarse proxy, as more detailed information about intervention content, dose, and treatment completion was unavailable. Given the absence of these data points and the modest change observed in loneliness over time, the present findings do not permit conclusions regarding the specific mechanisms by which loneliness may have been addressed during PTSD treatment or how treatment-specific processes (e.g. client expectations or therapeutic alliance) may have influenced these outcomes.

Future studies should distinguish between the outcomes of existing interventions or even categories of intervention (e.g. trauma- and non-trauma-focused, individual- and couple-/family-based), ideally over a longer period of assessment with a greater number of timepoints. Such efforts would (1) provide evidence for any treatment mechanisms that drive change, (2) inform approaches for tailoring interventions to improve both loneliness and PTSD symptom change outcomes, and (3) help elucidate whether/how these changes develop and are sustained over time. Studies might also consider applying more targeted and/or integrated methods (e.g. experimental designs and/or clinical or qualitative interviews in addition to self-reports) to evaluate their aims. Loneliness and PTSD treatment outcomes should be similarly investigated in samples comprised of nonveterans and/or more socio-demographically diverse veterans (e.g. rural, female, non-White, gender, or sexual minority), since the present study’s sample of predominantly male, White, combat-exposed, and urban-dwelling veterans limits the generalizability of its findings to other populations. Lastly, future studies may disentangle social loneliness (absence of a broader social network) from emotional loneliness (absence of a close other; Walsh et al., 2025); the levels of these subtypes of loneliness differ as a function of age (Manoli et al., 2022), which emerged as a predictor of change in PTSD symptoms (both with or without loneliness in the model) in our sample.

### Clinical implications

The present findings suggest that loneliness is a clinically meaningful factor to consider in PTSD treatment among veterans. A growing body of research demonstrates that loneliness is both highly prevalent among individuals with mental health difficulties and amenable to psychological intervention. Cognitive–behavioral therapies have been found to produce small-to-moderate reductions in loneliness (Hickin et al., 2021), perhaps due, in part, to targeting of maladaptive social beliefs (Mann et al., 2017). A particularly scalable model, therapist-guided internet-based cognitive–behavioral therapy focused specifically on loneliness, led to significantly greater reductions in loneliness compared to interpersonal psychotherapy and waitlist control conditions, with concomitant improvements in quality of life and emotional distress (Käll et al., 2021). Another RCT showed that interpersonal therapy adapted to be trauma-focused led to greater improvements in loneliness than usual care (Duberstein et al., 2018). Together, these findings suggest that loneliness is a modifiable, clinically relevant construct that warrants direct assessment and intervention.

These findings further indicate that addressing loneliness directly may enhance PTSD treatment outcomes. Trauma-focused psychotherapies may reduce loneliness indirectly by targeting maladaptive beliefs and emotional numbing, particularly within the negative alterations in cognitions and mood and dysphoric arousal symptom clusters. However, given the minimal change in loneliness observed over time, adjunctive or integrated interventions that explicitly target social connection may be warranted. Group-based interventions, peer support programs, and treatments that focus on increasing perceived social support and belonging have shown promise in improving mental health outcomes among veterans and trauma-exposed populations (Keyan et al., 2024; Pfeiffer et al., 2011). Incorporating such approaches into PTSD care may help address residual loneliness, reduce suicide risk, and promote more comprehensive recovery among veterans seeking treatment. It is also important to note that loneliness may pose barriers to treatment initiation and recovery. Studies of patients with depression show that loneliness is associated with lower help-seeking intentions (Teo et al., 2018) suggesting that loneliness may interfere with treatment engagement.

Beyond loneliness, veterans seeking PTSD treatment report poorer social functioning than other treatment-seeking veterans (Tsai et al., 2012) and often identify interpersonal difficulties as desired targets for intervention (Rosen et al., 2013). Meta-analytic findings show that having more perceived social support (which, along with objective social isolation, is conceptually distinct from loneliness but has been much more consistently researched) is associated with a better response to trauma-focused psychotherapies for PTSD (medium effect size; Keyan et al., 2024).

Taken together, these studies suggest that veterans with PTSD are often experiencing loneliness and are interested in addressing this issue in treatment, and that having stronger relationships benefits veterans during treatment. Routine assessment of loneliness at treatment intake may therefore enhance case conceptualization and help identify veterans at risk for persistent distress, even in the context of symptom improvement. Incorporating loneliness assessment is particularly relevant given evidence that perceived social disconnection and negative interpersonal cognitions are central to both PTSD maintenance (Ehlers & Clark, 2000) and suicidal desire through thwarted belongingness and perceived burdensomeness (Van Orden et al., 2010). Monitoring loneliness during treatment may also provide clinically relevant information beyond symptom change alone, especially for veterans who show improvement in PTSD symptoms but continue to experience impaired quality of life.

## Conclusion

Loneliness is common among veterans entering PTSD specialty care and is associated with greater symptom severity and distinct patterns of symptom change over time. Although lonelier veterans demonstrate greater improvement in PTSD symptoms related to negative alterations in cognitions and mood and dysphoric arousal, they remain more symptomatic, highlighting loneliness as a clinically significant factor in PTSD recovery. Integrating loneliness assessment and intervention into PTSD treatment planning may represent an important target for improving outcomes and promoting recovery among veterans.

## Supporting information

10.1017/S0033291726105157.sm001 Sippel et al. supplementary material Sippel et al. supplementary material

## Supplementary material

To view supplementary material for this article, please visit http://doi.org/10.1017/S0033291726105157.

## Long descriptions

### Table 1. Long description

The table is divided into two columns: Baseline characteristics and Frequency (percentage adjusted for missing n super a through h).

Demographic breakdown:

* Sex: 1,150 total. Male 883 (76.8 percent), Female 267 (23.2 percent).

* Race and ethnicity: 1,155 total. White 590 (51.1 percent), Black/African American 269 (23.3 percent), More than one race 116 (10.0 percent), Hispanic/Latinx 100 (8.7 percent), Unknown 37 (3.2 percent), Asian American 19 (1.7 percent), American Indian and Alaska Native 12 (1.0 percent), Native Hawaiian and Pacific Islander 12 (1.0 percent).

* Age: 1,148 total. The largest group is 36 to 45 (25.2 percent), followed by 56 to 65 (21.0 percent), 46 to 55 (19.8 percent), 26 to 35 (14.9 percent), 66 to 75 (11.2 percent), 76 plus (5.0 percent), and 18 to 25 (3.1 percent).

* Marital status: 1,121 total. Married 576 (48.6 percent), Not married 545 (48.6 percent).

* Employment: 1,153 total. Employed 552 (47.9 percent), Not employed 601 (52.1 percent).

* Rurality: 1,112 total. Urban 839 (75.5 percent), Rural plus 273 (24.6 percent).

* Combat history: 1,155 total. Yes 746 (64.6 percent), No 409 (35.4 percent).

Encounters section (winsorized, n = 1,145):

* Total Mental Health: Mean (S D) is 9.88 (9.93). 1 or more encounters: 1,022 (88.48 percent). Greater than 2 encounters: 973 (84.24 percent).

* Individual psychotherapy: 1 or more encounters: 835 (72.29 percent). Greater than 2 encounters: 754 (65.28 percent).

* Group psychotherapy: 1 or more encounters: 263 (22.77 percent). Greater than 2 encounters: 229 (19.83 percent).

* Medication Management: 1 or more encounters: 578 (50.04 percent). Greater than 2 encounters: 439 (38.01 percent).

Navigate back to Table 1..

### Table 2. Long description

The table presents data for 14 measures across two timepoints.

Baseline Measures (1 to 7):

* 1. Loneliness: M = 5.47, S D = 2.91.

* 2. Total P C L-5: M = 44.95, S D = 16.72; correlation with Loneliness is .44.

* 3. Reexperiencing: M = 2.29, S D = 1.01; correlation with Loneliness is .33.

* 4. Avoidance: M = 2.65, S D = 1.17; correlation with Loneliness is .29.

* 5. N A C M: M = 2.15, S D = 0.97; correlation with Loneliness is .49.

* 6. Anxious: M = 2.68, S D = 1.14; correlation with Loneliness is .22.

* 7. Dysphoric: M = 1.98, S D = 0.88; correlation with Loneliness is .38.

Follow-up Measures (8 to 14):

* 8. Loneliness: M = 4.32, S D = 2.92; correlation with baseline Loneliness is .43.

* 9. Total P C L-5: M = 36.17, S D = 20.73; correlation with follow-up Loneliness is .54.

* 10. Reexperiencing: M = 1.92, S D = 1.17; correlation with follow-up Loneliness is .43.

* 11. Avoidance: M = 2.15, S D = 1.35; correlation with follow-up Loneliness is .46.

* 12. N A C M: M = 1.71, S D = 1.12; correlation with follow-up Loneliness is .57.

* 13. Anxious: M = 2.15, S D = 1.32; correlation with follow-up Loneliness is .35.

* 14. Dysphoric: M = 1.56, S D = 1.01; correlation with follow-up Loneliness is .49.

All correlations mentioned are significant at p < .0001. N A C M stands for Negative Alterations to Cognitions and Mood.

Navigate back to Table 2..

### Table 3. Long description

The table contains 11 columns: Measure, Baseline (M, S D, Range, n), Follow-up (M, S D, Range, n), t-value, and Effect size d.

* Loneliness: Baseline M 5.47, S D 2.91, Range 0–12, n 1,146. Follow-up M 4.32, S D 2.92, Range 0–12, n 714. t 9.65, d .3945.

* P C L-5 factors:

* Reexperiencing: Baseline M 2.29, S D 1.01, Range 0–4, n 1,142. Follow-up M 1.92, S D 1.17, Range 0–4, n 714. t 9.09, d .3411.

* Avoidance: Baseline M 2.65, S D 1.17, Range 0–4, n 1,140. Follow-up M 2.15, S D 1.35, Range 0–4, n 712. t 9.20, d .3981.

* N A C M: Baseline M 2.15, S D 0.97, Range 0–4, n 1,141. Follow-up M 1.71, S D 1.12, Range 0–4, n 711. t 9.98, d .4159.

* Anxious: Baseline M 2.68, S D 1.14, Range 0–4, n 1,140. Follow-up M 2.15, S D 1.32, Range 0–4, n 710. t 10.91, d .4267.

* Dysphoric: Baseline M 1.98, S D 0.88, Range 0–4, n 1,140. Follow-up M 1.56, S D 1.01, Range 0–4, n 710. t 10.74, d .4422.

* P C L-5 total scores: Baseline M 44.95, S D 16.72, Range 0–80, n 1,099. Follow-up M 36.17, S D 20.73, Range 0–80, n 692. t 11.97, d .4658.

All t-values are significant at p < .0001.

Navigate back to Table 3..

### Table 4. Long description

The table presents two steps of a hierarchical regression. The columns for both Step 1 (covariates only) and Step 2 (covariates plus loneliness) include beta, S E, t, and p values.

* Predictors and Step 1 results:

- Intercept: beta 17.50, p .0468.

- Age groups (relative to 18–25): 26–35 (beta -5.27), 36–45 (beta -1.23), 46–55 (beta -4.73), 56–65 (beta -6.40), 66–75 (beta -8.95, p .0468), 76+ (beta -11.43, p .0262).

- Race and ethnicity (relative to white): American Indian and Alaska Native (beta 7.85), Asian American (beta -.996), Black/African American (beta 3.40), Native Hawaiian and Pacific Islander (beta -6.43), Hispanic/Latinx (beta -4.25), Other (beta 3.40), Unknown (beta -.735).

- Marital status: beta 1.24.

- Sex: beta 3.68, p .0490.

- Rurality: beta 3.92, p .0294.

- Combat history: beta 1.81.

- Total encounters: beta .081.

- Model R super 2: .052.

* Step 2 results (Loneliness added):

- Intercept: beta 14.94, p .0941.

- Loneliness, baseline: beta .423, S E .253, t 1.67, p .0956.

- Demographic coefficients remain largely similar to Step 1, with Rurality remaining significant (beta 4.01, p .0262) and Age 76+ remaining significant (beta -11.14, p .0304).

- Model R super 2: .056.

Note: Positive beta values indicate greater P T S D symptom improvement.

Navigate back to Table 4..

### Figure 1. Long description

A forest plot with a vertical axis labeled P T S D symptom-change outcome and a horizontal axis labeled Standardized Beta for Baseline Loneliness ranging from negative 0.10 to 0.20. A dashed vertical line marks the zero point. Six outcomes are plotted from top to bottom with blue circular point estimates and brown horizontal error bars representing confidence intervals.

* Total P C L dash 5 change: Point estimate at approximately 0.07 with error bars from negative 0.01 to 0.14.

* Reexperiencing change: Point estimate at approximately 0.07 with error bars from negative 0.01 to 0.14.

* Avoidance change: Point estimate at approximately 0.035 with error bars from negative 0.04 to 0.11.

* N A C M change: Point estimate at approximately 0.09 with error bars from 0.01 to 0.16.

* Anxious arousal change: Point estimate at approximately 0.025 with error bars from negative 0.05 to 0.10.

* Dysphoric arousal change: Point estimate at approximately 0.09 with error bars from 0.01 to 0.16.

Navigate back to Figure 1..

### Table 5. Long description

The table contains 12 rows of dependent variables analyzed across 8 statistical columns: D F, Sum of squares Model, Mean square Model, F Value, Pr > F, Error mean square, D F error, and Corrected total. All rows have a D F of 2.

* Baseline P C L 5 total: F Value 111.74, Pr < .0001.

* Follow-up P C L 5 total: F Value 28.48, Pr < .0001.

* Baseline reexperiencing: F Value 64, Pr < .0001.

* Follow-up reexperiencing: F Value 16.45, Pr < .0001.

* Baseline avoidance: F Value 43.45, Pr < .0001.

* Follow-up avoidance: F Value 18.79, Pr < .0001.

* Baseline N A C M: F Value 145.15, Pr < .0001.

* Follow-up N A C M: F Value 40.63, Pr < .0001.

* Baseline anxious arousal: F Value 23.59, Pr < .0001.

* Follow-up anxious arousal: F Value 7.97, Pr 0.0004.

* Baseline dysphoric arousal: F Value 80.65, Pr < .0001.

* Follow-up dysphoric arousal: F Value 22.64, Pr < .0001.

A note indicates that Tukey’s H S D post hoc tests showed significant differences between all loneliness groups for all variables except Follow-up Anxious Arousal, where the Medium and High groups did not differ.

Navigate back to Table 5..

### Figure 2. Long description

A line graph with the Y axis labeled Total P C L 5 Score ranging from 0 to 60 in increments of 10. The X axis is labeled Timepoint with two categories: Baseline and Follow-Up. Three distinct lines represent loneliness tertiles:

* High Loneliness: Represented by a dotted line with triangle markers. It starts at approximately 53 at Baseline and shows a linear decrease to approximately 43 at Follow-Up.

* Medium Loneliness: Represented by a dashed line with square markers. It starts at approximately 44 at Baseline and shows a linear decrease to approximately 35 at Follow-Up.

* Low Loneliness: Represented by a solid line with open circle markers. It starts at approximately 36 at Baseline and shows a linear decrease to approximately 29 at Follow-Up.

All three groups show a parallel downward trend over time, with the High Loneliness group consistently maintaining the highest P C L 5 scores and the Low Loneliness group maintaining the lowest.

Navigate back to Figure 2..
