# Supplementary material for: Loneliness as a predictor of posttraumatic stress disorder symptom change among treatment-seeking US military veterans
Source: Psychol Med. 2026 Jul 22;56:e233. doi: 10.1017/S0033291726105157 (PMC13439258; doi:10.1017/S0033291726105157)
Supplement: Supplementary file 1 [file S0033291726105157sup001.docx]

**Supplemental Materials**

**Supplemental Table A.**

*Hierarchical Regression Predicting Change in the* ***Reexperiencing Symptoms*** *on the PCL-5, Based on Loneliness, Demographics, and* ***Total Encounters***

| Predictor | Step 1 (covariates only) | | | | Step 2 (covariates plus loneliness) | | | |
| --- | --- | --- | --- | --- | --- | --- | --- | --- |
|  | β | SE | *t* | *p* | β | SE | *t* | *p* |
| **Intercept** | .954 | .481 | 1.98 | .0477 | .805 | .488 | 1.65 | .0995 |
| **Age group** |  |  |  |  |  |  |  |  |
| 26-35 | -.395 | .267 | -1.48 | .1404 | -.392 | .267 | -1.47 | .1427 |
| 36-45 | -.235 | .257 | -.92 | .3600 | -.246 | .257 | -.96 | .3388 |
| 46-55 | -.319 | .260 | -1.23 | .2196 | -.326 | .259 | -1.26 | .2088 |
| 56-65 | -.470 | .256 | -1.84 | .0666 | -.466 | .256 | -1.82 | .0685 |
| 66-75 | -.468 | .271 | -1.73 | .0843 | -.460 | .271 | -1.70 | .0897 |
| 76+ | -.701 | .304 | -2.31 | .0214 | -.684 | .304 | -2.25 | .0248 |
| **Race and ethnicity** |  |  |  |  |  |  |  |  |
| American Indian and Alaska Native | .343 | .420 | .82 | .4141 | .343 | .419 | .82 | .4135 |
| Asian American | .011 | .291 | .04 | .9689 | .058 | .300 | .19 | .8470 |
| Black/African American | .112 | .113 | .99 | .3241 | .108 | .114 | .95 | .3427 |
| Native Hawaiian and Pacific Islander | -.237 | .446 | -.53 | .5953 | -.254 | .446 | -.57 | .5697 |
| Hispanic/Latinx | -.265 | .171 | -1.55 | .1225 | -.243 | .171 | -1.42 | .1558 |
| More than one race | .080 | .260 | .31 | .7571 | .093 | .259 | .36 | .7196 |
| Unknown | -.101 | .155 | -.65 | .5178 | -.070 | .156 | -.45 | .6528 |
| **Marital status** | -.026 | .092 | -.29 | .7753 | -.006 | .094 | -.06 | .9483 |
| **Sex (Male)** | .173 | .110 | 1.56 | .1182 | .163 | .111 | 1.46 | .1440 |
| **Rurality (Urban)** | .126 | .106 | 1.19 | .2360 | .127 | .106 | 1.20 | .2308 |
| **Combat history (Yes)** | .115 | .094 | 1.22 | .2225 | .113 | .095 | 1.20 | .2305 |
| **Total encounters** | .006 | .004 | 1.51 | .1312 | .006 | .004 | 1.39 | .1652 |
| **Loneliness, baseline** | — | — | — | — | .026 | .015 | 1.75 | .0808 |
| **Model R^2^** |  |  |  | .033 |  |  |  | .036 |

Note. Positive β values indicate greater PTSD symptom improvement (i.e., larger reduction in PCL-5 scores). Step 1 model: N = 708, F(18, 689) = 1.29, p = .1888, R² = .033, adjusted R² = .007. Step 2 model: N = 703, F(19, 683) = 1.36, p = .1382, R² = .036, adjusted R² = .010. Reference groups are the omitted categories in the dummy-coded model. Encounter variables were winsorized.

**Supplemental Table B.**

*Hierarchical Regression Predicting Change in the* ***Avoidance*** *Symptoms on the PCL-5, Based on Loneliness, Demographics, and* ***Total Encounters***

| Predictor | Step 1 (covariates only) | | | | Step 2 (covariates plus loneliness) | | | | |
| --- | --- | --- | --- | --- | --- | --- | --- | --- | --- |
|  | β | SE | *t* | *p* | β | SE | *t* | *p* |  |
| **Intercept** | .513 | .616 | .83 | .4046 | .390 | .626 | .62 | .5332 |  |
| **Age group** |  |  |  |  |  |  |  |  |  |
| 26-35 | -.014 | .342 | -.04 | .9677 | -.014 | .342 | -.04 | .9678 |  |
| 36-45 | .077 | .329 | .24 | .8142 | .076 | .330 | .23 | .8178 |  |
| 46-55 | -.130 | .333 | -.39 | .6958 | -.134 | .333 | -.40 | .6883 |  |
| 56-65 | -.375 | .327 | -1.15 | .2517 | -.349 | .328 | -1.06 | .2875 |  |
| 66-75 | -.113 | .347 | -.33 | .7452 | -.104 | .347 | -.30 | .7643 |  |
| 76+ | -.217 | .389 | -.56 | .5771 | -.201 | .390 | -.52 | .6052 |  |
| **Race and ethnicity** |  |  |  |  |  |  |  |  |  |
| American Indian and Alaska Native | .162 | .537 | .30 | .7630 | .143 | .538 | .27 | .7906 |  |
| Asian American | .104 | .373 | .28 | .7807 | .177 | .384 | .46 | .6453 |  |
| Black/African American | .048 | .145 | .33 | .7396 | .037 | .146 | .25 | .7996 |  |
| Native Hawaiian and Pacific Islander | -.319 | .571 | -.56 | .5765 | -.333 | .571 | -.58 | .5601 |  |
| Hispanic/Latinx | -.165 | .221 | -.75 | .4548 | -.158 | .221 | -.71 | .4764 |  |
| More than one race | .375 | .332 | 1.13 | .2587 | .380 | .333 | 1.14 | .2540 |  |
| Unknown | .060 | .199 | .30 | .7647 | .063 | .200 | .32 | .7516 |  |
| **Marital status** | .151 | .118 | 1.27 | .2030 | .171 | .120 | 1.43 | .1543 |  |
| **Sex (Male)** | .418 | .141 | 2.95 | .0032 | .442 | .143 | 3.10 | .0020 |  |
| **Rurality (Urban)** | .105 | .136 | .77 | .4388 | .114 | .136 | .84 | .4026 |  |
| **Combat history (Yes)** | .106 | .121 | .87 | .3820 | .096 | .121 | .79 | .4301 |  |
| **Total encounters** | .003 | .005 | .67 | .5016 | .003 | .005 | .58 | .5615 |  |
| **Loneliness, baseline** | — | — | — | — | .017 | .019 | .91 | .3647 |  |
| **Model R^2^** |  |  |  | .031 |  |  |  | .033 |  |

Note. Positive β values indicate greater PTSD symptom improvement (i.e., larger reduction in PCL-5 scores). Step 1 model: N = 705, F(18, 686) = 1.24, p = .2246, R² = .031, adjusted R² = .006. Step 2 model: N = 700, F(19, 680) = 1.23, p = .2270, R² = .033, adjusted R² = .006. Reference groups are the omitted categories in the dummy-coded model. Encounter variables were winsorized.

**Supplemental Table C.**

*Hierarchical Regression Predicting Change in the* ***NACM*** *Symptoms on the PCL-5, Based on Loneliness, Demographics, and* ***Total Encounters***

| Predictor | Step 1 (covariates only) | | | | Step 2 (covariates plus loneliness) | | | |
| --- | --- | --- | --- | --- | --- | --- | --- | --- |
|  | β | SE | *t* | *p* | β | SE | *t* | *p* |
| **Intercept** | .544 | .445 | 1.22 | .2216 | .352 | .451 | .78 | .4362 |
| **Age group** |  |  |  |  |  |  |  |  |
| 26-35 | -.161 | .247 | -.65 | .5151 | -.151 | .247 | -.61 | .5423 |
| 36-45 | .041 | .238 | .17 | .8642 | .039 | .238 | .16 | .8690 |
| 46-55 | -.224 | .240 | -.93 | .3526 | -.226 | .240 | -.94 | .3458 |
| 56-65 | -.262 | .237 | -1.11 | .2683 | -.255 | .236 | -1.08 | .2805 |
| 66-75 | -.454 | .251 | -1.81 | .0708 | -.438 | .250 | -1.75 | .0805 |
| 76+ | -.414 | .281 | -1.47 | .1414 | -.387 | .281 | -1.38 | .1691 |
| **Race and ethnicity** |  |  |  |  |  |  |  |  |
| American Indian and Alaska Native | .078 | .388 | .20 | .8412 | .071 | .388 | .18 | .8537 |
| Asian American | -.252 | .270 | -.93 | .3509 | -.250 | .277 | -.90 | .3661 |
| Black/African American | .098 | .105 | .93 | .3519 | .087 | .105 | .83 | .4094 |
| Native Hawaiian and Pacific Islander | -.463 | .412 | -1.12 | .2618 | -.492 | .412 | -1.19 | .2328 |
| Hispanic/Latinx | -.172 | .160 | -1.08 | .2807 | -.153 | .160 | -.96 | .3396 |
| More than one race | .042 | .240 | .17 | .8617 | .056 | .240 | .23 | .8157 |
| Unknown | .013 | .144 | .09 | .9288 | .011 | .144 | .08 | .9384 |
| **Marital status** | -.015 | .086 | -.17 | .8639 | .019 | .087 | .21 | .8307 |
| **Sex (Male)** | .136 | .103 | 1.33 | .1847 | .148 | .103 | 1.43 | .1520 |
| **Rurality (Urban)** | .219 | .098 | 2.23 | .0261 | .223 | .098 | 2.27 | .0234 |
| **Combat history (Yes)** | .044 | .087 | .50 | .6167 | .026 | .087 | .29 | .7687 |
| **Total encounters** | .004 | .004 | 1.04 | .3000 | .003 | .004 | .94 | .3468 |
| **Loneliness, baseline** | — | — | — | — | .032 | .014 | 2.30 | .0220 |
| **Model R^2^** |  |  |  | .041 |  |  |  | .049 |

Note. Positive β values indicate greater PTSD symptom improvement (i.e., larger reduction in PCL-5 scores). Step 1 model: N = 705, F(18, 686) = 1.63, p = .0473, R² = .041, adjusted R² = .016. Step 2 model: N = 700, F(19, 680) = 1.84, p = .0160, R² = .049, adjusted R² = .022. Reference groups are the omitted categories in the dummy-coded model. Encounter variables were winsorized.

**Supplemental Table D.**

*Hierarchical Regression Predicting Change in the* ***Anxious Arousal*** *Symptoms on the PCL-5, Based on Loneliness, Demographics, and* ***Total Encounters***

| Predictor | Step 1 (covariates only) | | | | Step 2 (covariates plus loneliness) | | | |
| --- | --- | --- | --- | --- | --- | --- | --- | --- |
|  | β | SE | *t* | *p* | β | SE | *t* | *p* |
| **Intercept** | .847 | .545 | 1.55 | .1207 | .760 | .555 | 1.37 | .1717 |
| **Age group** |  |  |  |  |  |  |  |  |
| 26-35 | -.233 | .303 | -.77 | .4415 | -.228 | .304 | -.75 | .4542 |
| 36-45 | .040 | .291 | .14 | .8920 | .035 | .292 | .12 | .9046 |
| 46-55 | -.308 | .295 | -1.04 | .2968 | -.309 | .295 | -1.05 | .2959 |
| 56-65 | -.319 | .290 | -1.10 | .2709 | -.310 | .291 | -1.07 | .2865 |
| 66-75 | -.447 | .307 | -1.46 | .1461 | -.441 | .308 | -1.43 | .1531 |
| 76+ | -.701 | .345 | -2.04 | .0422 | -.689 | .346 | -1.99 | .0465 |
| **Race and ethnicity** |  |  |  |  |  |  |  |  |
| American Indian and Alaska Native | .232 | .476 | .49 | .6257 | .225 | .477 | .47 | .6372 |
| Asian American | -.243 | .330 | -.74 | .4625 | -.226 | .341 | -.66 | .5073 |
| Black/African American | .161 | .129 | 1.26 | .2099 | .157 | .130 | 1.21 | .2262 |
| Native Hawaiian and Pacific Islander | -.310 | .505 | -.61 | .5404 | -.322 | .507 | -.64 | .5249 |
| Hispanic/Latinx | -.173 | .196 | -.88 | .3769 | -.165 | .196 | -.84 | .4025 |
| More than one race | .344 | .294 | 1.17 | .2424 | .352 | .295 | 1.19 | .2331 |
| Unknown | -.132 | .176 | -.75 | .4555 | -.146 | .177 | -.82 | .4108 |
| **Marital status** | .002 | .105 | .02 | .9832 | .022 | .107 | .20 | .8387 |
| **Sex (Male)** | .135 | .126 | 1.07 | .2834 | .148 | .127 | 1.17 | .2441 |
| **Rurality (Urban)** | .197 | .121 | 1.64 | .1023 | .202 | .121 | 1.67 | .0959 |
| **Combat history (Yes)** | .184 | .107 | 1.71 | .0868 | .172 | .108 | 1.60 | .1104 |
| **Total encounters** | .001 | .005 | .31 | .7595 | .001 | .005 | .27 | .7901 |
| **Loneliness, baseline** | — | — | — | — | .013 | .017 | .77 | .4416 |
| **Model R^2^** |  |  |  | .041 |  |  |  | .042 |

Note. Positive β values indicate greater PTSD symptom improvement (i.e., larger reduction in PCL-5 scores). Step 1 model: N = 704, F(18, 685) = 1.62, p = .0505, R² = .041, adjusted R² = .015. Step 2 model: N = 699, F(19, 679) = 1.55, p = .0637, R² = .042, adjusted R² = .015. Reference groups are the omitted categories in the dummy-coded model. Encounter variables were winsorized.

**Supplemental Table E.**

*Hierarchical Regression Predicting Change in the* ***Dysphoric Arousal*** *Symptoms on the PCL-5, Based on Loneliness and* ***Total Encounters***

| Predictor | Step 1 (covariates only) | | | | Step 2 (covariates plus loneliness) | | | |
| --- | --- | --- | --- | --- | --- | --- | --- | --- |
|  | β | SE | *t* | *p* | β | SE | *t* | *p* |
| **Intercept** | .268 | .407 | .66 | .5103 | .099 | .410 | .24 | .8086 |
| **Age group** |  |  |  |  |  |  |  |  |
| 26-35 | -.282 | .226 | -1.25 | .2129 | -.268 | .225 | -1.19 | .2329 |
| 36-45 | -.111 | .217 | -.51 | .6105 | -.115 | .216 | -.53 | .5956 |
| 46-55 | -.143 | .220 | -.65 | .5142 | -.142 | .218 | -.65 | .5148 |
| 56-65 | -.331 | .216 | -1.53 | .1260 | -.323 | .215 | -1.50 | .1337 |
| 66-75 | -.501 | .229 | -2.19 | .0292 | -.483 | .228 | -2.12 | .0342 |
| 76+ | -.523 | .257 | -2.04 | .0421 | -.497 | .255 | -1.95 | .0521 |
| **Race and ethnicity** |  |  |  |  |  |  |  |  |
| American Indian and Alaska Native | -.240 | .355 | -.68 | .4990 | -.249 | .352 | -.71 | .4809 |
| Asian American | -.156 | .247 | -.63 | .5268 | -.084 | .252 | -.33 | .7386 |
| Black/African American | .097 | .096 | 1.01 | .3139 | .076 | .096 | .79 | .4308 |
| Native Hawaiian and Pacific Islander | -.237 | .377 | -.63 | .5294 | -.267 | .375 | -.71 | .4768 |
| Hispanic/Latinx | -.253 | .146 | -1.73 | .0837 | -.238 | .145 | -1.64 | .1013 |
| More than one race | .241 | .219 | 1.10 | .2731 | .250 | .218 | 1.15 | .2512 |
| Unknown | -.205 | .131 | -1.56 | .1192 | -.240 | .131 | -1.83 | .0679 |
| **Marital status** | .093 | .078 | 1.19 | .2349 | .117 | .079 | 1.48 | .1387 |
| **Sex (Male)** | .099 | .094 | 1.06 | .2903 | .106 | .094 | 1.13 | .2607 |
| **Rurality (Urban)** | .167 | .090 | 1.86 | .0633 | .170 | .090 | 1.89 | .0586 |
| **Combat history (Yes)** | .128 | .080 | 1.60 | .1102 | .108 | .080 | 1.36 | .1740 |
| **Total encounters** | .001 | .003 | .37 | .7110 | .001 | .003 | .24 | .8108 |
| **Loneliness, baseline** | — | — | — | — | .029 | .013 | 2.27 | .0234 |
| **Model R^2^** |  |  |  | .045 |  |  |  | .053 |

Note. Positive β values indicate greater PTSD symptom improvement (i.e., larger reduction in PCL-5 scores). Step 1 model: N = 704, F(18, 685) = 1.80, p = .0215, R² = .045, adjusted R² = .020. Step 2 model: N = 699, F(19, 679) = 2.02, p = .0063, R² = .053, adjusted R² = .027. Reference groups are the omitted categories in the dummy-coded model. Encounter variables were winsorized.

**Supplemental Table F.**

*Hierarchical Regression Predicting* ***Total PCL-5*** *Score Change, Based on Loneliness, Demographic Covariates, and* ***Individual Psychotherapy Encounters*** *at Baseline*

| Predictor | Step 1 (covariates only) | | | | Step 2 (covariates plus loneliness) | | | |
| --- | --- | --- | --- | --- | --- | --- | --- | --- |
|  | β | SE | t | β | SE | SE | β | SE |
| **Intercept** | 17.43 | 8.79 | 1.98 | .0479 | 15.09 | 8.92 | 1.69 | .0914 |
| **Age group** |  |  |  |  |  |  |  |  |
| 26-35 | -5.12 | 4.39 | -1.16 | .2446 | -5.00 | 4.40 | -1.14 | .2561 |
| 36-45 | -1.20 | 4.24 | -.28 | .7770 | -1.32 | 4.25 | -.31 | .7565 |
| 46-55 | -4.55 | 4.27 | -1.07 | .2865 | -4.61 | 4.27 | -1.08 | .2801 |
| 56-65 | -6.01 | 4.21 | -1.43 | .1536 | -5.96 | 4.22 | -1.41 | .1582 |
| 66-75 | -8.63 | 4.51 | -1.91 | .0559 | -8.50 | 4.51 | -1.89 | .0598 |
| 76+ | -10.90 | 5.14 | -2.12 | .0346 | -10.68 | 5.15 | -2.07 | .0385 |
| **Race and ethnicity** |  |  |  |  |  |  |  |  |
| American Indian and Alaska Native | 8.28 | 7.91 | 1.05 | .2959 | 8.06 | 7.92 | 1.02 | .3089 |
| Asian American | -.713 | 5.09 | -.14 | .8886 | .050 | 5.26 | .01 | .9924 |
| Black/African American | 3.40 | 1.95 | 1.74 | .0818 | 3.17 | 1.96 | 1.61 | .1068 |
| Native Hawaiian and Pacific Islander | -6.34 | 7.31 | -.87 | .3863 | -6.69 | 7.32 | -.91 | .3607 |
| Hispanic/Latinx | -4.30 | 2.88 | -1.49 | .1362 | -4.13 | 2.89 | -1.43 | .1526 |
| More than one race | 3.37 | 4.25 | .79 | .4288 | 3.51 | 4.26 | .83 | .4095 |
| Unknown | -.475 | 2.61 | -.18 | .8559 | -.556 | 2.63 | -.21 | .8324 |
| **Marital status** | 1.12 | 1.57 | .71 | .4760 | 1.46 | 1.59 | .92 | .3574 |
| **Sex (Male)** | 3.59 | 1.87 | 1.92 | .0556 | 3.57 | 1.88 | 1.90 | .0575 |
| **Rurality (Urban)** | 3.68 | 1.80 | 2.04 | .0416 | 3.77 | 1.81 | 2.09 | .0373 |
| **Combat history (Yes)** | 1.67 | 1.60 | 1.04 | .2988 | 1.50 | 1.61 | .93 | .3508 |
| **Total encounters** | .197 | .115 | 1.71 | .0875 | .182 | .116 | 1.57 | .1167 |
| **Loneliness, baseline** | — | — | — | — | .388 | .255 | 1.52 | .1295 |
| **Model R^2^** |  |  |  | .052 |  |  |  | .056 |

Note. Positive β values indicate greater PTSD symptom improvement (i.e., larger reduction in PCL-5 scores). Step 1 model: N = 655, F(18, 636) = 1.95, p = .0105, R² = .052, adjusted R² = .025. Step 2 model: N = 652, F(19, 632) = 1.96, p = .0089, R² = .056, adjusted R² = .027. Reference groups are the omitted categories in the dummy-coded model. Encounter variables were winsorized.

**Supplemental Table G.**

*Hierarchical Regression Predicting* ***Reexperiencing*** *Symptom Change, Based on Loneliness, Demographic Covariates, and* ***Individual Psychotherapy Encounters*** *at Baseline*

| Predictor | Step 1 (covariates only) | | | | Step 2 (covariates plus loneliness) | | | |
| --- | --- | --- | --- | --- | --- | --- | --- | --- |
|  | β | SE |  | β | SE |  | β | SE |
| **Intercept** | .928 | .481 | 1.93 | .0538 | .807 | .488 | 1.65 | .0990 |
| **Age group** |  |  |  |  |  |  |  |  |
| 26-35 | -.382 | .267 | -1.43 | .1528 | -.382 | .267 | -1.43 | .1530 |
| 36-45 | -.226 | .257 | -.88 | .3788 | -.237 | .257 | -.92 | .3575 |
| 46-55 | -.306 | .259 | -1.18 | .2377 | -.314 | .259 | -1.21 | .2265 |
| 56-65 | -.440 | .255 | -1.72 | .0856 | -.440 | .256 | -1.72 | .0856 |
| 66-75 | -.443 | .271 | -1.63 | .1027 | -.440 | .271 | -1.62 | .1052 |
| 76+ | -.662 | .304 | -2.18 | .0296 | -.652 | .304 | -2.14 | .0323 |
| **Race and ethnicity** |  |  |  |  |  |  |  |  |
| American Indian and Alaska Native | .376 | .419 | .90 | .3696 | .373 | .419 | .89 | .3740 |
| Asian American | .037 | .291 | .13 | .8996 | .079 | .300 | .26 | .7918 |
| Black/African American | .117 | .114 | 1.02 | .3061 | .113 | .114 | .98 | .3253 |
| Native Hawaiian and Pacific Islander | -.226 | .445 | -.51 | .6121 | -.241 | .445 | -.54 | .5887 |
| Hispanic/Latinx | -.262 | .172 | -1.52 | .1278 | -.247 | .172 | -1.43 | .1521 |
| More than one race | .079 | .259 | .30 | .7614 | .089 | .259 | .34 | .7307 |
| Unknown | -.078 | .155 | -.50 | .6179 | -.052 | .156 | -.33 | .7394 |
| **Marital status** | -.038 | .092 | -.41 | .6788 | -.020 | .094 | -.21 | .8317 |
| **Sex (Male)** | .159 | .111 | 1.43 | .1527 | .151 | .112 | 1.35 | .1767 |
| **Rurality (Urban)** | .113 | .106 | 1.06 | .2886 | .113 | .106 | 1.06 | .2882 |
| **Combat history (Yes)** | .105 | .094 | 1.11 | .2670 | .105 | .095 | 1.11 | .2694 |
| **Total encounters** | .018 | .007 | 2.61 | .0094 | .016 | .007 | 2.34 | .0195 |
| **Loneliness, baseline** | — | — | — | — | .022 | .015 | 1.48 | .1402 |
| **Model R^2^** |  |  |  | .037 |  |  |  | .039 |

Note. Positive β values indicate greater PTSD symptom improvement (i.e., larger reduction in PCL-5 scores). Step 1 model: N = 701, F(18, 682) = 1.47, p = .0921, R² = .037, adjusted R² = .012. Step 2 model: N = 696, F(19, 676) = 1.46, p = .0932, R² = .039, adjusted R² = .012. Reference groups are the omitted categories in the dummy-coded model. Encounter variables were winsorized.

**Supplemental Table H.**

*Hierarchical Regression Predicting* ***Avoidance*** *Symptom Change, Based on Loneliness, Demographic Covariates, and* ***Individual Psychotherapy Encounters*** *at Baseline*

| Predictor | Step 1 (covariates plus encounters) | | | | Step 2 (covariates plus encounters and loneliness) | | | |
| --- | --- | --- | --- | --- | --- | --- | --- | --- |
|  | β | SE | t | p | β | SE | t | p |
| **Intercept** | .503 | .617 | .81 | .4157 | .400 | .628 | .64 | .5241 |
| **Age group** |  |  |  |  |  |  |  |  |
| 26-35 | -.005 | .343 | -.02 | .9873 | -.007 | .343 | -.02 | .9830 |
| 36-45 | .082 | .330 | .25 | .8035 | .081 | .331 | .24 | .8072 |
| 46-55 | -.118 | .333 | -.35 | .7234 | -.122 | .333 | -.37 | .7142 |
| 56-65 | -.348 | .328 | -1.06 | .2888 | -.324 | .329 | -.98 | .3251 |
| 66-75 | -.103 | .348 | -.30 | .7675 | -.098 | .349 | -.28 | .7781 |
| 76+ | -.187 | .390 | -.48 | .6315 | -.176 | .391 | -.45 | .6524 |
| **Race and ethnicity** |  |  |  |  |  |  |  |  |
| American Indian and Alaska Native | .183 | .538 | .34 | .7334 | .164 | .539 | .30 | .7612 |
| Asian American | .124 | .374 | .33 | .7396 | .196 | .385 | .51 | .6109 |
| Black/African American | .050 | .146 | .34 | .7329 | .040 | .147 | .27 | .7862 |
| Native Hawaiian and Pacific Islander | -.310 | .572 | -.54 | .5884 | -.321 | .573 | -.56 | .5748 |
| Hispanic/Latinx | -.171 | .223 | -.77 | .4426 | -.168 | .223 | -.75 | .4532 |
| More than one race | .377 | .332 | 1.13 | .2574 | .380 | .333 | 1.14 | .2541 |
| Unknown | .075 | .200 | .37 | .7088 | .076 | .201 | .38 | .7063 |
| **Marital status** | .141 | .119 | 1.18 | .2366 | .160 | .121 | 1.32 | .1870 |
| **Sex (Male)** | .412 | .142 | 2.89 | .0040 | .438 | .144 | 3.05 | .0024 |
| **Rurality (Urban)** | .088 | .137 | .64 | .5206 | .096 | .137 | .70 | .4832 |
| **Combat history (Yes)** | .088 | .122 | .73 | .4676 | .080 | .122 | .65 | .5131 |
| **Total encounters** | .011 | .009 | 1.19 | .2329 | .010 | .009 | 1.09 | .2782 |
| **Loneliness, baseline** | — | — | — | — | .014 | .019 | .73 | .4671 |
| **Model R^2^** |  |  |  | .032 |  |  |  | .033 |

Note. Positive β values indicate greater PTSD symptom improvement (i.e., larger reduction in PCL-5 scores). Step 1 model: N = 698, F(18, 679) = 1.23, p = .2299, R² = .032, adjusted R² = .006. Step 2 model: N = 693, F(19, 673) = 1.20, p = .2468, R² = .033, adjusted R² = .006. Reference groups are the omitted categories in the dummy-coded model. Encounter variables were winsorized.

**Supplemental Table I.**

*Hierarchical Regression Predicting* ***NACM*** *Symptom Change, Based on Loneliness, Demographic Covariates, and* ***Individual Psychotherapy Encounters*** *at Baseline*

| Predictor | Step 1 (covariates plus encounters) | | | | Step 2 (covariates plus encounters and loneliness) | | | |
| --- | --- | --- | --- | --- | --- | --- | --- | --- |
|  | β | SE | t | p | β | SE | t | p |
| **Intercept** | .539 | .446 | 1.21 | .2279 | .356 | .453 | .79 | .4314 |
| **Age group** |  |  |  |  |  |  |  |  |
| 26-35 | -.154 | .248 | -.62 | .5339 | -.146 | .248 | -.59 | .5566 |
| 36-45 | .045 | .239 | .19 | .8512 | .043 | .238 | .18 | .8578 |
| 46-55 | -.215 | .241 | -.89 | .3722 | -.219 | .240 | -.91 | .3631 |
| 56-65 | -.247 | .237 | -1.04 | .2980 | -.243 | .237 | -1.03 | .3050 |
| 66-75 | -.438 | .252 | -1.74 | .0829 | -.426 | .252 | -1.69 | .0906 |
| 76+ | -.398 | .282 | -1.41 | .1588 | -.375 | .282 | -1.33 | .1840 |
| **Race and ethnicity** |  |  |  |  |  |  |  |  |
| American Indian and Alaska Native | .087 | .389 | .22 | .8223 | .079 | .388 | .20 | .8383 |
| Asian American | -.244 | .270 | -.90 | .3674 | -.245 | .278 | -.88 | .3790 |
| Black/African American | .097 | .106 | .92 | .3587 | .086 | .106 | .81 | .4177 |
| Native Hawaiian and Pacific Islander | -.463 | .413 | -1.12 | .2634 | -.491 | .413 | -1.19 | .2347 |
| Hispanic/Latinx | -.177 | .161 | -1.10 | .2713 | -.163 | .161 | -1.01 | .3135 |
| More than one race | .036 | .240 | .15 | .8803 | .049 | .240 | .21 | .8372 |
| Unknown | .021 | .144 | .15 | .8821 | .017 | .145 | .12 | .9061 |
| **Marital status** | -.021 | .086 | -.24 | .8068 | .013 | .087 | .14 | .8849 |
| **Sex (Male)** | .124 | .103 | 1.20 | .2286 | .138 | .104 | 1.33 | .1848 |
| **Rurality (Urban)** | .207 | .099 | 2.10 | .0363 | .211 | .099 | 2.13 | .0331 |
| **Combat history (Yes)** | .039 | .088 | .44 | .6590 | .022 | .088 | .25 | .8035 |
| **Total encounters** | .009 | .006 | 1.36 | .1757 | .008 | .006 | 1.19 | .2342 |
| **Loneliness, baseline** | — | — | — | — | .030 | .014 | 2.17 | .0305 |
| **Model R^2^** |  |  |  | .040 |  |  |  | .047 |

Note. Positive β values indicate greater PTSD symptom improvement (i.e., larger reduction in PCL-5 scores). Step 1 model: N = 698, F(18, 679) = 1.58, p = .0596, R² = .040, adjusted R² = .015. Step 2 model: N = 693, F(19, 673) = 1.76, p = .0242, R² = .047, adjusted R² = .020. Reference groups are the omitted categories in the dummy-coded model. Encounter variables were winsorized.

**Supplemental Table J.**

*Hierarchical Regression Predicting* ***Anxious*** ***Arousal*** *Symptom Change, Based on Loneliness, Demographic Covariates, and* ***Individual Psychotherapy Encounters*** *at Baseline*

| Predictor | Step 1 (covariates plus encounters) | | | | Step 2 (covariates plus encounters and loneliness) | | | |
| --- | --- | --- | --- | --- | --- | --- | --- | --- |
|  | β | SE | t | p | β | SE | t | p |
| **Intercept** | .833 | .547 | 1.52 | .1279 | .765 | .557 | 1.37 | .1698 |
| **Age group** |  |  |  |  |  |  |  |  |
| 26-35 | -.224 | .303 | -.74 | .4614 | -.219 | .304 | -.72 | .4713 |
| 36-45 | .031 | .292 | .11 | .9147 | .027 | .293 | .09 | .9277 |
| 46-55 | -.301 | .295 | -1.02 | .3073 | -.303 | .295 | -1.02 | .3061 |
| 56-65 | -.292 | .291 | -1.00 | .3156 | -.283 | .292 | -.97 | .3320 |
| 66-75 | -.405 | .309 | -1.31 | .1892 | -.402 | .309 | -1.30 | .1944 |
| 76+ | -.682 | .345 | -1.97 | .0489 | -.673 | .347 | -1.94 | .0526 |
| **Race and ethnicity** |  |  |  |  |  |  |  |  |
| American Indian and Alaska Native | .247 | .476 | .52 | .6046 | .240 | .478 | .50 | .6155 |
| Asian American | -.226 | .331 | -.68 | .4952 | -.209 | .342 | -.61 | .5417 |
| Black/African American | .168 | .130 | 1.29 | .1960 | .165 | .131 | 1.26 | .2068 |
| Native Hawaiian and Pacific Islander | -.305 | .506 | -.60 | .5468 | -.315 | .508 | -.62 | .5351 |
| Hispanic/Latinx | -.140 | .197 | -.71 | .4796 | -.134 | .198 | -.68 | .4982 |
| More than one race | .352 | .294 | 1.20 | .2325 | .359 | .295 | 1.21 | .2249 |
| Unknown | -.117 | .177 | -.66 | .5096 | -.133 | .178 | -.74 | .4570 |
| **Marital status** | -.007 | .105 | -.07 | .9436 | .010 | .107 | .09 | .9255 |
| **Sex (Male)** | .125 | .126 | .98 | .3251 | .139 | .128 | 1.09 | .2781 |
| **Rurality (Urban)** | .197 | .121 | 1.62 | .1049 | .201 | .122 | 1.65 | .0996 |
| **Combat history (Yes)** | .181 | .108 | 1.68 | .0925 | .172 | .108 | 1.58 | .1140 |
| **Total encounters** | .005 | .008 | .68 | .4958 | .005 | .008 | .65 | .5178 |
| **Loneliness, baseline** | — | — | — | — | .010 | .017 | .57 | .5684 |
| **Model R^2^** |  |  |  | .038 |  |  |  | .038 |

Note. Positive β values indicate greater PTSD symptom improvement (i.e., larger reduction in PCL-5 scores). Step 1 model: N = 697, F(18, 678) = 1.49, p = .0869, R² = .038, adjusted R² = .013. Step 2 model: N = 692, F(19, 672) = 1.41, p = .1122, R² = .038, adjusted R² = .011. Reference groups are the omitted categories in the dummy-coded model. Encounter variables were winsorized.

**Supplemental Table K.**

*Hierarchical Regression Predicting* ***Dysphoric*** ***Arousal*** *Symptom Change, Based on Loneliness, Demographic Covariates, and* ***Individual Psychotherapy Encounters*** *at Baseline*

| Predictor | Step 1 (covariates plus encounters) | | | | Step 2 (covariates plus encounters and loneliness) | | | |
| --- | --- | --- | --- | --- | --- | --- | --- | --- |
|  | β | SE | t | p | β | SE | t | p |
| **Intercept** | .268 | .408 | .66 | .5118 | .096 | .412 | .23 | .8164 |
| **Age group** |  |  |  |  |  |  |  |  |
| 26-35 | -.282 | .227 | -1.24 | .2140 | -.269 | .225 | -1.20 | .2322 |
| 36-45 | -.123 | .218 | -.56 | .5730 | -.128 | .217 | -.59 | .5548 |
| 46-55 | -.143 | .220 | -.65 | .5147 | -.144 | .218 | -.66 | .5087 |
| 56-65 | -.328 | .217 | -1.51 | .1307 | -.322 | .216 | -1.50 | .1353 |
| 66-75 | -.509 | .230 | -2.21 | .0276 | -.494 | .229 | -2.16 | .0312 |
| 76+ | -.522 | .258 | -2.02 | .0433 | -.497 | .256 | -1.94 | .0528 |
| **Race and ethnicity** |  |  |  |  |  |  |  |  |
| American Indian and Alaska Native | -.236 | .356 | -.66 | .5077 | -.242 | .353 | -.69 | .4925 |
| Asian American | -.148 | .247 | -.60 | .5485 | -.076 | .253 | -.30 | .7622 |
| Black/African American | .096 | .097 | .99 | .3236 | .074 | .097 | .77 | .4409 |
| Native Hawaiian and Pacific Islander | -.236 | .378 | -.62 | .5324 | -.266 | .375 | -.71 | .4793 |
| Hispanic/Latinx | -.267 | .147 | -1.81 | .0706 | -.254 | .147 | -1.74 | .0832 |
| More than one race | .244 | .220 | 1.11 | .2668 | .255 | .218 | 1.17 | .2428 |
| Unknown | -.198 | .132 | -1.50 | .1346 | -.233 | .132 | -1.77 | .0770 |
| **Marital status** | .100 | .079 | 1.27 | .2047 | .126 | .079 | 1.59 | .1122 |
| **Sex (Male)** | .090 | .094 | .95 | .3418 | .096 | .094 | 1.02 | .3076 |
| **Rurality (Urban)** | .162 | .091 | 1.78 | .0748 | .165 | .090 | 1.83 | .0683 |
| **Combat history (Yes)** | .122 | .080 | 1.52 | .1284 | .104 | .080 | 1.29 | .1958 |
| **Total encounters** | .003 | .006 | .52 | .6007 | .003 | .006 | .47 | .6420 |
| **Loneliness, baseline** | — | — | — | — | .029 | .013 | 2.29 | .0221 |
| **Model R^2^** |  |  |  | .045 |  |  |  | .053 |

Note. Positive β values indicate greater PTSD symptom improvement (i.e., larger reduction in PCL-5 scores). Step 1 model: N = 697, F(18, 678) = 1.76, p = .0265, R² = .045, adjusted R² = .019. Step 2 model: N = 692, F(19, 672) = 1.99, p = .0073, R² = .053, adjusted R² = .027. Reference groups are the omitted categories in the dummy-coded model. Encounter variables were winsorized.

**Supplemental Table L.**

*Hierarchical Regression Predicting* ***Total*** *PCL-5 Score Change, Based on Loneliness, Demographic Covariates, and* ***Group Psychotherapy Encounters*** *at Baseline*

| Predictor | Step 1 (covariates plus encounters) | | | | Step 2 (covariates plus encounters and loneliness) | | | |
| --- | --- | --- | --- | --- | --- | --- | --- | --- |
|  | β | SE | t | p | β | SE | t | p |
| **Intercept** | 17.86 | 8.81 | 2.03 | .0430 | 15.30 | 8.94 | 1.71 | .0874 |
| **Age group** |  |  |  |  |  |  |  |  |
| 26-35 | -5.16 | 4.40 | -1.17 | .2419 | -5.03 | 4.41 | -1.14 | .2540 |
| 36-45 | -1.27 | 4.25 | -.30 | .7658 | -1.38 | 4.26 | -.32 | .7464 |
| 46-55 | -4.28 | 4.28 | -1.00 | .3170 | -4.37 | 4.28 | -1.02 | .3073 |
| 56-65 | -6.18 | 4.22 | -1.47 | .1432 | -6.10 | 4.22 | -1.45 | .1489 |
| 66-75 | -8.63 | 4.52 | -1.91 | .0567 | -8.49 | 4.52 | -1.88 | .0609 |
| 76+ | -11.29 | 5.15 | -2.19 | .0288 | -11.01 | 5.16 | -2.14 | .0330 |
| **Race and ethnicity** |  |  |  |  |  |  |  |  |
| American Indian and Alaska Native | 7.15 | 7.91 | .90 | .3663 | 7.01 | 7.91 | .89 | .3761 |
| Asian American | -1.15 | 5.09 | -.23 | .8209 | -.310 | 5.27 | -.06 | .9530 |
| Black/African American | 3.08 | 1.95 | 1.58 | .1151 | 2.85 | 1.96 | 1.45 | .1463 |
| Native Hawaiian and Pacific Islander | -6.67 | 7.32 | -.91 | .3628 | -7.02 | 7.33 | -.96 | .3382 |
| Hispanic/Latinx | -4.70 | 2.89 | -1.62 | .1048 | -4.50 | 2.90 | -1.55 | .1213 |
| More than one race | 3.08 | 4.27 | .72 | .4710 | 3.25 | 4.27 | .76 | .4470 |
| Unknown | -.709 | 2.61 | -.27 | .7862 | -.754 | 2.63 | -.29 | .7742 |
| **Marital status** | 1.12 | 1.57 | .71 | .4780 | 1.48 | 1.59 | .93 | .3525 |
| **Sex (Male)** | 3.84 | 1.87 | 2.05 | .0408 | 3.81 | 1.88 | 2.02 | .0434 |
| **Rurality (Urban)** | 3.52 | 1.81 | 1.95 | .0517 | 3.64 | 1.81 | 2.01 | .0453 |
| **Combat history (Yes)** | 1.57 | 1.61 | .98 | .3296 | 1.40 | 1.61 | .87 | .3846 |
| **Total encounters** | -.065 | .131 | -.50 | .6204 | -.062 | .131 | -.47 | .6350 |
| **Loneliness, baseline** | — | — | — | — | .417 | .255 | 1.64 | .1021 |
| **Model R^2^** |  |  |  | .048 |  |  |  | .052 |

Note. Positive β values indicate greater PTSD symptom improvement (i.e., larger reduction in PCL-5 scores). Step 1 model: N = 655, F(18, 636) = 1.80, p = .0225, R² = .048, adjusted R² = .021. Step 2 model: N = 652, F(19, 632) = 1.83, p = .0169, R² = .052, adjusted R² = .024. Reference groups are the omitted categories in the dummy-coded model. Encounter variables were winsorized.

**Supplemental Table M.**

*Hierarchical Regression Predicting* ***Reexperiencing*** *Symptom Change, Based on Loneliness, Demographic Covariates, and* ***Group Psychotherapy Encounters*** *at Baseline*

| Predictor | Step 1 (covariates plus encounters) | | | | Step 2 (covariates plus encounters and loneliness) | | | |
| --- | --- | --- | --- | --- | --- | --- | --- | --- |
|  | β | SE | t | p | β | SE | t | p |
| **Intercept** | .988 | .482 | 2.05 | .0408 | .845 | .490 | 1.73 | .0847 |
| **Age group** |  |  |  |  |  |  |  |  |
| 26-35 | -.383 | .268 | -1.43 | .1534 | -.383 | .268 | -1.43 | .1537 |
| 36-45 | -.227 | .258 | -.88 | .3790 | -.239 | .258 | -.93 | .3543 |
| 46-55 | -.283 | .260 | -1.09 | .2769 | -.294 | .260 | -1.13 | .2593 |
| 56-65 | -.450 | .257 | -1.75 | .0803 | -.449 | .257 | -1.75 | .0809 |
| 66-75 | -.448 | .272 | -1.65 | .0999 | -.444 | .272 | -1.63 | .1031 |
| 76+ | -.688 | .305 | -2.26 | .0242 | -.674 | .305 | -2.21 | .0273 |
| **Race and ethnicity** |  |  |  |  |  |  |  |  |
| American Indian and Alaska Native | .291 | .420 | .69 | .4882 | .296 | .420 | .70 | .4812 |
| Asian American | -.006 | .292 | -.02 | .9836 | .046 | .300 | .15 | .8789 |
| Black/African American | .089 | .114 | .78 | .4373 | .086 | .115 | .75 | .4513 |
| Native Hawaiian and Pacific Islander | -.252 | .447 | -.56 | .5727 | -.266 | .447 | -.60 | .5511 |
| Hispanic/Latinx | -.299 | .173 | -1.72 | .0853 | -.278 | .173 | -1.60 | .1094 |
| More than one race | .048 | .260 | .18 | .8540 | .063 | .260 | .24 | .8096 |
| Unknown | -.103 | .156 | -.66 | .5069 | -.073 | .157 | -.47 | .6403 |
| **Marital status** | -.042 | .093 | -.45 | .6500 | -.021 | .094 | -.23 | .8211 |
| **Sex (Male)** | .181 | .111 | 1.62 | .1048 | .170 | .112 | 1.51 | .1304 |
| **Rurality (Urban)** | .095 | .107 | .89 | .3757 | .098 | .107 | .91 | .3611 |
| **Combat history (Yes)** | .097 | .095 | 1.03 | .3045 | .098 | .095 | 1.03 | .3035 |
| **Total encounters** | -.008 | .008 | -1.01 | .3152 | -.007 | .008 | -.92 | .3591 |
| **Loneliness, baseline** | — | — | — | — | .025 | .015 | 1.65 | .1000 |
| **Model R^2^** |  |  |  | .029 |  |  |  | .033 |

Note. Positive β values indicate greater PTSD symptom improvement (i.e., larger reduction in PCL-5 scores). Step 1 model: N = 701, F(18, 682) = 1.14, p = .3034, R² = .029, adjusted R² = .004. Step 2 model: N = 696, F(19, 676) = 1.21, p = .2443, R² = .033, adjusted R² = .006. Reference groups are the omitted categories in the dummy-coded model. Encounter variables were winsorized.

**Supplemental Table N.**

*Hierarchical Regression Predicting* ***Avoidance*** *Symptom Change, Based on Loneliness, Demographic Covariates, and* ***Group Psychotherapy Encounters*** *at Baseline*

| Predictor | Step 1 (covariates plus encounters) | | | | Step 2 (covariates plus encounters and loneliness) | | | |
| --- | --- | --- | --- | --- | --- | --- | --- | --- |
|  | β | SE | t | p | β | SE | t | p |
| **Intercept** | .538 | .617 | .87 | .3836 | .423 | .628 | .67 | .5009 |
| **Age group** |  |  |  |  |  |  |  |  |
| 26-35 | -.007 | .343 | -.02 | .9832 | -.009 | .344 | -.03 | .9797 |
| 36-45 | .080 | .330 | .24 | .8089 | .078 | .331 | .23 | .8147 |
| 46-55 | -.105 | .333 | -.32 | .7519 | -.111 | .334 | -.33 | .7389 |
| 56-65 | -.356 | .329 | -1.08 | .2786 | -.331 | .329 | -1.01 | .3149 |
| 66-75 | -.107 | .349 | -.31 | .7586 | -.102 | .349 | -.29 | .7705 |
| 76+ | -.205 | .390 | -.52 | .6001 | -.191 | .391 | -.49 | .6249 |
| **Race and ethnicity** |  |  |  |  |  |  |  |  |
| American Indian and Alaska Native | .135 | .538 | .25 | .8022 | .119 | .538 | .22 | .8247 |
| Asian American | .100 | .374 | .27 | .7902 | .176 | .385 | .46 | .6472 |
| Black/African American | .034 | .146 | .23 | .8169 | .025 | .147 | .17 | .8670 |
| Native Hawaiian and Pacific Islander | -.326 | .572 | -.57 | .5693 | -.337 | .573 | -.59 | .5561 |
| Hispanic/Latinx | -.193 | .224 | -.87 | .3873 | -.187 | .224 | -.83 | .4049 |
| More than one race | .361 | .333 | 1.08 | .2792 | .366 | .334 | 1.10 | .2726 |
| Unknown | .059 | .200 | .30 | .7668 | .063 | .201 | .31 | .7539 |
| **Marital status** | .140 | .119 | 1.17 | .2409 | .160 | .121 | 1.32 | .1864 |
| **Sex (Male)** | .424 | .142 | 2.98 | .0030 | .448 | .144 | 3.12 | .0019 |
| **Rurality (Urban)** | .078 | .137 | .57 | .5672 | .088 | .137 | .64 | .5212 |
| **Combat history (Yes)** | .083 | .122 | .68 | .4936 | .075 | .122 | .62 | .5384 |
| **Total encounters** | -.003 | .010 | -.35 | .7283 | -.003 | .010 | -.32 | .7509 |
| **Loneliness, baseline** | — | — | — | — | .016 | .019 | .81 | .4159 |
| **Model R^2^** |  |  |  | .030 |  |  |  | .031 |

Note. Positive β values indicate greater PTSD symptom improvement (i.e., larger reduction in PCL-5 scores). Step 1 model: N = 698, F(18, 679) = 1.16, p = .2934, R² = .030, adjusted R² = .004. Step 2 model: N = 693, F(19, 673) = 1.15, p = .2995, R² = .031, adjusted R² = .004. Reference groups are the omitted categories in the dummy-coded model. Encounter variables were winsorized.

**Supplemental Table O.**

*Hierarchical Regression Predicting* ***NACM*** *Symptom Change, Based on Loneliness, Demographic Covariates, and* ***Group Psychotherapy Encounters*** *at Baseline*

| Predictor | Step 1 (covariates plus encounters) | | | | Step 2 (covariates plus encounters and loneliness) | | | |
| --- | --- | --- | --- | --- | --- | --- | --- | --- |
|  | β | SE | t | p | β | SE | t | p |
| **Intercept** | .567 | .446 | 1.27 | .2040 | .374 | .453 | .83 | .4088 |
| **Age group** |  |  |  |  |  |  |  |  |
| 26-35 | -.156 | .248 | -.63 | .5311 | -.147 | .248 | -.59 | .5535 |
| 36-45 | .043 | .239 | .18 | .8567 | .040 | .239 | .17 | .8667 |
| 46-55 | -.204 | .241 | -.85 | .3971 | -.211 | .241 | -.88 | .3818 |
| 56-65 | -.253 | .238 | -1.06 | .2874 | -.250 | .238 | -1.05 | .2937 |
| 66-75 | -.440 | .252 | -1.75 | .0814 | -.430 | .252 | -1.70 | .0888 |
| 76+ | -.412 | .282 | -1.46 | .1451 | -.387 | .282 | -1.37 | .1703 |
| **Race and ethnicity** |  |  |  |  |  |  |  |  |
| American Indian and Alaska Native | .047 | .389 | .12 | .9041 | .045 | .388 | .11 | .9086 |
| Asian American | -.264 | .270 | -.98 | .3285 | -.260 | .278 | -.94 | .3496 |
| Black/African American | .084 | .106 | .79 | .4286 | .074 | .106 | .70 | .4843 |
| Native Hawaiian and Pacific Islander | -.476 | .414 | -1.15 | .2504 | -.504 | .413 | -1.22 | .2234 |
| Hispanic/Latinx | -.196 | .162 | -1.21 | .2250 | -.177 | .162 | -1.10 | .2738 |
| More than one race | .022 | .241 | .09 | .9262 | .039 | .241 | .16 | .8714 |
| Unknown | .009 | .144 | .06 | .9508 | .007 | .145 | .05 | .9614 |
| **Marital status** | -.022 | .086 | -.26 | .7976 | .013 | .087 | .15 | .8806 |
| **Sex (Male)** | .135 | .103 | 1.31 | .1911 | .146 | .104 | 1.41 | .1597 |
| **Rurality (Urban)** | .199 | .099 | 2.01 | .0445 | .205 | .099 | 2.07 | .0389 |
| **Combat history (Yes)** | .035 | .088 | .39 | .6936 | .018 | .088 | .21 | .8354 |
| **Total encounters** | -.003 | .007 | -.45 | .6495 | -.002 | .007 | -.31 | .7534 |
| **Loneliness, baseline** | — | — | — | — | .032 | .014 | 2.27 | .0238 |
| **Model R^2^** |  |  |  | .038 |  |  |  | .045 |

Note. Positive β values indicate greater PTSD symptom improvement (i.e., larger reduction in PCL-5 scores). Step 1 model: N = 698, F(18, 679) = 1.48, p = .0885, R² = .038, adjusted R² = .012. Step 2 model: N = 693, F(19, 673) = 1.68, p = .0344, R² = .045, adjusted R² = .018. Reference groups are the omitted categories in the dummy-coded model. Encounter variables were winsorized.

**Supplemental Table P.**

*Hierarchical Regression Predicting* ***Anxious*** ***Arousal*** *Symptom Change, Based on Loneliness, Demographic Covariates, and* ***Group Psychotherapy Encounters*** *at Baseline*

| Predictor | Step 1 (covariates plus encounters) | | | | Step 2 (covariates plus encounters and loneliness) | | | |
| --- | --- | --- | --- | --- | --- | --- | --- | --- |
|  | β | SE | t | p | β | SE | t | p |
| **Intercept** | .851 | .546 | 1.56 | .1194 | .777 | .557 | 1.40 | .1630 |
| **Age group** |  |  |  |  |  |  |  |  |
| 26-35 | -.225 | .304 | -.74 | .4589 | -.221 | .304 | -.73 | .4687 |
| 36-45 | .029 | .292 | .10 | .9197 | .024 | .293 | .08 | .9348 |
| 46-55 | -.296 | .295 | -1.00 | .3164 | -.299 | .296 | -1.01 | .3132 |
| 56-65 | -.297 | .291 | -1.02 | .3078 | -.289 | .292 | -.99 | .3230 |
| 66-75 | -.408 | .309 | -1.32 | .1865 | -.405 | .310 | -1.31 | .1913 |
| 76+ | -.691 | .345 | -2.00 | .0458 | -.682 | .347 | -1.97 | .0494 |
| **Race and ethnicity** |  |  |  |  |  |  |  |  |
| American Indian and Alaska Native | .224 | .476 | .47 | .6381 | .219 | .477 | .46 | .6465 |
| Asian American | -.238 | .331 | -.72 | .4722 | -.218 | .341 | -.64 | .5224 |
| Black/African American | .160 | .129 | 1.24 | .2162 | .158 | .130 | 1.21 | .2264 |
| Native Hawaiian and Pacific Islander | -.313 | .506 | -.62 | .5365 | -.323 | .508 | -.64 | .5246 |
| Hispanic/Latinx | -.149 | .198 | -.75 | .4507 | -.142 | .199 | -.71 | .4750 |
| More than one race | .345 | .295 | 1.17 | .2420 | .354 | .296 | 1.20 | .2320 |
| Unknown | -.124 | .177 | -.70 | .4815 | -.139 | .178 | -.78 | .4340 |
| **Marital status** | -.008 | .106 | -.07 | .9413 | .011 | .107 | .10 | .9198 |
| **Sex (Male)** | .131 | .126 | 1.03 | .3024 | .144 | .128 | 1.12 | .2614 |
| **Rurality (Urban)** | .192 | .121 | 1.59 | .1134 | .197 | .122 | 1.62 | .1062 |
| **Combat history (Yes)** | .179 | .108 | 1.66 | .0970 | .169 | .108 | 1.56 | .1190 |
| **Total encounters** | -.001 | .009 | -.11 | .9102 | -.000 | .009 | -.05 | .9562 |
| **Loneliness, baseline** | — | — | — | — | .011 | .017 | .62 | .5327 |
| **Model R^2^** |  |  |  | .037 |  |  |  | .038 |

Note. Positive β values indicate greater PTSD symptom improvement (i.e., larger reduction in PCL-5 scores). Step 1 model: N = 697, F(18, 678) = 1.46, p = .0966, R² = .037, adjusted R² = .012. Step 2 model: N = 692, F(19, 672) = 1.39, p = .1230, R² = .038, adjusted R² = .011. Reference groups are the omitted categories in the dummy-coded model. Encounter variables were winsorized.

**Supplemental Table Q.**

*Hierarchical Regression Predicting* ***Dysphoric*** ***Arousal*** *Symptom Change, Based on Loneliness, Demographic Covariates, and* ***Group Psychotherapy Encounters*** *at Baseline*

| Predictor | Step 1 (covariates plus encounters) | | | | Step 2 (covariates plus encounters and loneliness) | | | |
| --- | --- | --- | --- | --- | --- | --- | --- | --- |
|  | β | SE | t | p | β | SE | t | p |
| **Intercept** | .278 | .408 | .68 | .4956 | .103 | .411 | .25 | .8023 |
| **Age group** |  |  |  |  |  |  |  |  |
| 26-35 | -.280 | .227 | -1.24 | .2164 | -.268 | .225 | -1.19 | .2344 |
| 36-45 | -.122 | .218 | -.56 | .5769 | -.127 | .217 | -.59 | .5578 |
| 46-55 | -.135 | .220 | -.61 | .5393 | -.137 | .219 | -.63 | .5304 |
| 56-65 | -.325 | .217 | -1.50 | .1342 | -.320 | .216 | -1.48 | .1387 |
| 66-75 | -.506 | .230 | -2.19 | .0286 | -.491 | .229 | -2.15 | .0322 |
| 76+ | -.523 | .258 | -2.03 | .0429 | -.498 | .256 | -1.94 | .0524 |
| **Race and ethnicity** |  |  |  |  |  |  |  |  |
| American Indian and Alaska Native | -.256 | .355 | -.72 | .4711 | -.261 | .353 | -.74 | .4600 |
| Asian American | -.157 | .247 | -.63 | .5258 | -.083 | .252 | -.33 | .7435 |
| Black/African American | .089 | .097 | .92 | .3585 | .068 | .096 | .71 | .4799 |
| Native Hawaiian and Pacific Islander | -.241 | .378 | -.64 | .5234 | -.270 | .375 | -.72 | .4714 |
| Hispanic/Latinx | -.280 | .148 | -1.89 | .0586 | -.265 | .147 | -1.81 | .0711 |
| More than one race | .234 | .220 | 1.06 | .2885 | .246 | .218 | 1.12 | .2610 |
| Unknown | -.202 | .132 | -1.53 | .1262 | -.237 | .132 | -1.80 | .0726 |
| **Marital status** | .098 | .079 | 1.24 | .2141 | .124 | .079 | 1.57 | .1175 |
| **Sex (Male)** | .096 | .094 | 1.02 | .3085 | .102 | .094 | 1.08 | .2811 |
| **Rurality (Urban)** | .156 | .091 | 1.72 | .0856 | .160 | .090 | 1.77 | .0770 |
| **Combat history (Yes)** | .120 | .080 | 1.50 | .1352 | .102 | .080 | 1.27 | .2038 |
| **Total encounters** | -.004 | .006 | -.69 | .4904 | -.004 | .006 | -.62 | .5355 |
| **Loneliness, baseline** | — | — | — | — | .030 | .013 | 2.32 | .0206 |
| **Model R^2^** |  |  |  | .045 |  |  |  | .054 |

Note. Positive β values indicate greater PTSD symptom improvement (i.e., larger reduction in PCL-5 scores). Step 1 model: N = 697, F(18, 678) = 1.77, p = .0251, R² = .045, adjusted R² = .020. Step 2 model: N = 692, F(19, 672) = 2.00, p = .0069, R² = .054, adjusted R² = .027. Reference groups are the omitted categories in the dummy-coded model. Encounter variables were winsorized.

**Supplemental Table R.**

*Hierarchical Regression Predicting* ***Total*** *PCL-5 Score Change, Based on Loneliness, Demographic Covariates, and* ***Medication*** ***Encounters*** *at Baseline*

| Predictor | Step 1 (covariates plus encounters) | | | | | Step 2 (covariates plus encounters and loneliness) | | | |
| --- | --- | --- | --- | --- | --- | --- | --- | --- | --- |
|  | β | SE | t | p | | β | SE | t | p |
| **Intercept** | 17.86 | 8.81 | 2.03 | .0431 | 15.30 | | 8.94 | 1.71 | .0876 |
| **Age group** |  |  |  |  |  | |  |  |  |
| 26-35 | -5.20 | 4.40 | -1.18 | .2383 | -5.04 | | 4.41 | -1.14 | .2533 |
| 36-45 | -1.30 | 4.26 | -.31 | .7596 | -1.38 | | 4.26 | -.32 | .7462 |
| 46-55 | -4.38 | 4.28 | -1.02 | .3066 | -4.40 | | 4.28 | -1.03 | .3048 |
| 56-65 | -6.27 | 4.22 | -1.49 | .1376 | -6.16 | | 4.22 | -1.46 | .1454 |
| 66-75 | -8.71 | 4.52 | -1.93 | .0542 | -8.55 | | 4.52 | -1.89 | .0591 |
| 76+ | -11.40 | 5.15 | -2.21 | .0272 | -11.11 | | 5.15 | -2.16 | .0314 |
| **Race and ethnicity** |  |  |  |  |  | |  |  |  |
| American Indian and Alaska Native | 7.28 | 7.92 | .92 | .3585 | 7.02 | | 7.92 | .89 | .3759 |
| Asian American | -1.14 | 5.10 | -.22 | .8234 | -.359 | | 5.27 | -.07 | .9457 |
| Black/African American | 3.14 | 1.95 | 1.61 | .1087 | 2.87 | | 1.96 | 1.47 | .1429 |
| Native Hawaiian and Pacific Islander | -6.65 | 7.33 | -.91 | .3646 | -7.07 | | 7.33 | -.96 | .3352 |
| Hispanic/Latinx | -4.58 | 2.89 | -1.59 | .1133 | -4.41 | | 2.89 | -1.52 | .1278 |
| More than one race | 3.19 | 4.27 | .75 | .4549 | 3.30 | | 4.27 | .77 | .4402 |
| Unknown | -.727 | 2.61 | -.28 | .7809 | -.796 | | 2.63 | -.30 | .7621 |
| **Marital status** | 1.14 | 1.58 | .72 | .4691 | 1.48 | | 1.60 | .93 | .3530 |
| **Sex (Male)** | 3.78 | 1.87 | 2.02 | .0437 | 3.76 | | 1.88 | 2.00 | .0457 |
| **Rurality (Urban)** | 3.57 | 1.81 | 1.97 | .0492 | 3.64 | | 1.82 | 2.00 | .0458 |
| **Combat history (Yes)** | 1.59 | 1.61 | .99 | .3247 | 1.39 | | 1.62 | .86 | .3906 |
| **Total encounters** | .008 | .288 | .03 | .9766 | -.073 | | .292 | -.25 | .8015 |
| **Loneliness, baseline** | — | — | — | — | .428 | | .257 | 1.67 | .0963 |
| **Model R^2^** |  |  |  | .048 |  | |  |  | .052 |

Note. Positive β values indicate greater PTSD symptom improvement (i.e., larger reduction in PCL-5 scores). Step 1 model: N = 655, F(18, 636) = 1.78, p = .0240, R² = .048, adjusted R² = .021. Step 2 model: N = 652, F(19, 632) = 1.82, p = .0176, R² = .052, adjusted R² = .023. Reference groups are the omitted categories in the dummy-coded model. Encounter variables were winsorized.

**Supplemental Table S.**

*Hierarchical Regression Predicting* ***Reexperiencing*** *Symptom Change, Based on Loneliness, Demographic Covariates, and* ***Medication Encounters*** *at Baseline*

| Predictor | Step 1 (covariates plus encounters) | | | | Step 2 (covariates plus encounters and loneliness) | | | |
| --- | --- | --- | --- | --- | --- | --- | --- | --- |
|  | β | SE | t | p | β | SE | t | p |
| **Intercept** | .991 | .482 | 2.05 | .0403 | .843 | .490 | 1.72 | .0856 |
| **Age group** |  |  |  |  |  |  |  |  |
| 26-35 | -.386 | .268 | -1.44 | .1501 | -.385 | .268 | -1.44 | .1513 |
| 36-45 | -.230 | .258 | -.89 | .3738 | -.242 | .258 | -.94 | .3493 |
| 46-55 | -.289 | .261 | -1.11 | .2677 | -.299 | .260 | -1.15 | .2515 |
| 56-65 | -.459 | .257 | -1.79 | .0742 | -.457 | .257 | -1.78 | .0752 |
| 66-75 | -.457 | .272 | -1.68 | .0940 | -.451 | .272 | -1.66 | .0977 |
| 76+ | -.698 | .305 | -2.29 | .0223 | -.683 | .305 | -2.24 | .0255 |
| **Race and ethnicity** |  |  |  |  |  |  |  |  |
| American Indian and Alaska Native | .299 | .421 | .71 | .4778 | .302 | .420 | .72 | .4726 |
| Asian American | -.007 | .292 | -.02 | .9820 | .044 | .301 | .15 | .8829 |
| Black/African American | .092 | .114 | .81 | .4204 | .089 | .115 | .78 | .4370 |
| Native Hawaiian and Pacific Islander | -.254 | .447 | -.57 | .5699 | -.269 | .447 | -.60 | .5470 |
| Hispanic/Latinx | -.286 | .173 | -1.66 | .0982 | -.266 | .173 | -1.54 | .1241 |
| More than one race | .058 | .260 | .22 | .8249 | .072 | .260 | .28 | .7825 |
| Unknown | -.105 | .156 | -.67 | .5005 | -.075 | .157 | -.48 | .6319 |
| **Marital status** | -.040 | .093 | -.43 | .6652 | -.019 | .094 | -.20 | .8432 |
| **Sex (Male)** | .176 | .111 | 1.58 | .1143 | .165 | .112 | 1.48 | .1403 |
| **Rurality (Urban)** | .098 | .107 | .91 | .3629 | .100 | .107 | .93 | .3502 |
| **Combat history (Yes)** | .097 | .095 | 1.03 | .3056 | .097 | .095 | 1.02 | .3079 |
| **Total encounters** | -.004 | .017 | -.26 | .7951 | -.004 | .018 | -.26 | .7973 |
| **Loneliness, baseline** | — | — | — | — | .026 | .015 | 1.69 | .0909 |
| **Model R^2^** |  |  |  | .028 |  |  |  | .032 |

Note. Positive β values indicate greater PTSD symptom improvement (i.e., larger reduction in PCL-5 scores). Step 1 model: N = 701, F(18, 682) = 1.09, p = .3567, R² = .028, adjusted R² = .002. Step 2 model: N = 696, F(19, 676) = 1.17, p = .2814, R² = .032, adjusted R² = .004. Reference groups are the omitted categories in the dummy-coded model. Encounter variables were winsorized.

**Supplemental Table T.**

*Hierarchical Regression Predicting* ***Avoidance*** *Symptom Change, Based on Loneliness, Demographic Covariates, and* ***Medication Encounters*** *at Baseline*

| Predictor | Step 1 (covariates plus encounters) | | | | Step 2 (covariates plus encounters and loneliness) | | | |
| --- | --- | --- | --- | --- | --- | --- | --- | --- |
|  | β | SE | t | p | β | SE | t | p |
| **Intercept** | .548 | .617 | .89 | .3749 | .421 | .628 | .67 | .5022 |
| **Age group** |  |  |  |  |  |  |  |  |
| 26-35 | -.005 | .343 | -.01 | .9884 | -.005 | .343 | -.01 | .9892 |
| 36-45 | .085 | .330 | .26 | .7973 | .084 | .331 | .25 | .8008 |
| 46-55 | -.095 | .334 | -.28 | .7767 | -.097 | .334 | -.29 | .7704 |
| 56-65 | -.354 | .328 | -1.08 | .2816 | -.327 | .329 | -.99 | .3204 |
| 66-75 | -.108 | .349 | -.31 | .7559 | -.101 | .349 | -.29 | .7719 |
| 76+ | -.211 | .390 | -.54 | .5892 | -.195 | .391 | -.50 | .6171 |
| **Race and ethnicity** |  |  |  |  |  |  |  |  |
| American Indian and Alaska Native | .116 | .538 | .22 | .8289 | .096 | .539 | .18 | .8590 |
| Asian American | .088 | .374 | .24 | .8142 | .164 | .385 | .42 | .6711 |
| Black/African American | .031 | .146 | .21 | .8346 | .019 | .147 | .13 | .8990 |
| Native Hawaiian and Pacific Islander | -.337 | .572 | -.59 | .5560 | -.353 | .573 | -.62 | .5380 |
| Hispanic/Latinx | -.195 | .223 | -.87 | .3827 | -.189 | .223 | -.85 | .3981 |
| More than one race | .353 | .333 | 1.06 | .2899 | .356 | .333 | 1.07 | .2856 |
| Unknown | .056 | .200 | .28 | .7772 | .058 | .201 | .29 | .7724 |
| **Marital status** | .135 | .119 | 1.13 | .2596 | .156 | .121 | 1.29 | .1989 |
| **Sex (Male)** | .424 | .142 | 2.98 | .0030 | .449 | .144 | 3.13 | .0018 |
| **Rurality (Urban)** | .070 | .137 | .51 | .6122 | .078 | .138 | .56 | .5725 |
| **Combat history (Yes)** | .078 | .122 | .64 | .5201 | .068 | .123 | .55 | .5793 |
| **Total encounters** | -.017 | .022 | -.77 | .4389 | -.020 | .022 | -.90 | .3689 |
| **Loneliness, baseline** | — | — | — | — | .018 | .019 | .92 | .3570 |
| **Model R^2^** |  |  |  | .030 |  |  |  | .032 |

Note. Positive β values indicate greater PTSD symptom improvement (i.e., larger reduction in PCL-5 scores). Step 1 model: N = 698, F(18, 679) = 1.18, p = .2688, R² = .030, adjusted R² = .005. Step 2 model: N = 693, F(19, 673) = 1.18, p = .2641, R² = .032, adjusted R² = .005. Reference groups are the omitted categories in the dummy-coded model. Encounter variables were winsorized.

**Supplemental Table U.**

*Hierarchical Regression Predicting* ***NACM*** *Symptom Change, Based on Loneliness, Demographic Covariates, and* ***Medication Encounters*** *at Baseline*

| Predictor | Step 1 (covariates plus encounters) | | | | Step 2 (covariates plus encounters and loneliness) | | | |
| --- | --- | --- | --- | --- | --- | --- | --- | --- |
|  | β | SE | t | p | β | SE | t | p |
| **Intercept** | .563 | .446 | 1.26 | .2075 | .374 | .453 | .83 | .4094 |
| **Age group** |  |  |  |  |  |  |  |  |
| 26-35 | -.159 | .248 | -.64 | .5210 | -.149 | .248 | -.60 | .5474 |
| 36-45 | .038 | .239 | .16 | .8738 | .037 | .239 | .16 | .8761 |
| 46-55 | -.216 | .241 | -.89 | .3720 | -.217 | .241 | -.90 | .3685 |
| 56-65 | -.261 | .238 | -1.10 | .2716 | -.255 | .237 | -1.07 | .2840 |
| 66-75 | -.446 | .252 | -1.77 | .0777 | -.433 | .252 | -1.72 | .0861 |
| 76+ | -.415 | .282 | -1.47 | .1419 | -.390 | .282 | -1.38 | .1673 |
| **Race and ethnicity** |  |  |  |  |  |  |  |  |
| American Indian and Alaska Native | .064 | .389 | .17 | .8688 | .054 | .389 | .14 | .8895 |
| Asian American | -.257 | .271 | -.95 | .3422 | -.257 | .278 | -.92 | .3553 |
| Black/African American | .088 | .106 | .84 | .4040 | .077 | .106 | .73 | .4676 |
| Native Hawaiian and Pacific Islander | -.470 | .414 | -1.14 | .2565 | -.501 | .413 | -1.21 | .2264 |
| Hispanic/Latinx | -.187 | .161 | -1.16 | .2469 | -.171 | .161 | -1.06 | .2880 |
| More than one race | .035 | .241 | .14 | .8861 | .046 | .241 | .19 | .8491 |
| Unknown | .010 | .144 | .07 | .9475 | .008 | .145 | .05 | .9586 |
| **Marital status** | -.017 | .086 | -.20 | .8413 | .016 | .087 | .18 | .8587 |
| **Sex (Male)** | .132 | .103 | 1.27 | .2029 | .144 | .104 | 1.39 | .1658 |
| **Rurality (Urban)** | .207 | .099 | 2.08 | .0376 | .209 | .099 | 2.10 | .0359 |
| **Combat history (Yes)** | .038 | .088 | .43 | .6672 | .020 | .088 | .23 | .8203 |
| **Total encounters** | .008 | .016 | .51 | .6115 | .004 | .016 | .23 | .8204 |
| **Loneliness, baseline** | — | — | — | — | .031 | .014 | 2.24 | .0257 |
| **Model R^2^** |  |  |  | .038 |  |  |  | .045 |

Note. Positive β values indicate greater PTSD symptom improvement (i.e., larger reduction in PCL-5 scores). Step 1 model: N = 698, F(18, 679) = 1.49, p = .0874, R² = .038, adjusted R² = .012. Step 2 model: N = 693, F(19, 673) = 1.68, p = .0348, R² = .045, adjusted R² = .018. Reference groups are the omitted categories in the dummy-coded model. Encounter variables were winsorized.

**Supplemental Table V.**

*Hierarchical Regression Predicting* ***Anxious*** ***Arousal*** *Symptom Change, Based on Loneliness, Demographic Covariates, and* ***Medication Encounters*** *at Baseline*

| Predictor | Step 1 (covariates plus encounters) | | | | Step 2 (covariates plus encounters and loneliness) | | | |
| --- | --- | --- | --- | --- | --- | --- | --- | --- |
|  | β | SE | t | p | β | SE | t | p |
| **Intercept** | .849 | .546 | 1.55 | .1205 | .777 | .557 | 1.40 | .1630 |
| **Age group** |  |  |  |  |  |  |  |  |
| 26-35 | -.227 | .304 | -.75 | .4557 | -.221 | .305 | -.73 | .4677 |
| 36-45 | .027 | .292 | .09 | .9261 | .023 | .293 | .08 | .9367 |
| 46-55 | -.301 | .295 | -1.02 | .3087 | -.300 | .296 | -1.01 | .3113 |
| 56-65 | -.300 | .291 | -1.03 | .3021 | -.290 | .292 | -.99 | .3209 |
| 66-75 | -.410 | .309 | -1.33 | .1842 | -.406 | .310 | -1.31 | .1903 |
| 76+ | -.692 | .345 | -2.00 | .0454 | -.683 | .346 | -1.97 | .0491 |
| **Race and ethnicity** |  |  |  |  |  |  |  |  |
| American Indian and Alaska Native | .232 | .476 | .49 | .6266 | .221 | .478 | .46 | .6435 |
| Asian American | -.234 | .331 | -.71 | .4792 | -.218 | .342 | -.64 | .5241 |
| Black/African American | .162 | .129 | 1.25 | .2107 | .159 | .131 | 1.22 | .2247 |
| Native Hawaiian and Pacific Islander | -.310 | .506 | -.61 | .5405 | -.323 | .508 | -.63 | .5258 |
| Hispanic/Latinx | -.146 | .197 | -.74 | .4608 | -.141 | .198 | -.71 | .4777 |
| More than one race | .350 | .295 | 1.19 | .2350 | .355 | .296 | 1.20 | .2299 |
| Unknown | -.124 | .177 | -.70 | .4829 | -.139 | .178 | -.78 | .4346 |
| **Marital status** | -.006 | .106 | -.05 | .9580 | .011 | .107 | .11 | .9157 |
| **Sex (Male)** | .129 | .126 | 1.02 | .3070 | .143 | .128 | 1.12 | .2626 |
| **Rurality (Urban)** | .196 | .122 | 1.61 | .1083 | .198 | .122 | 1.62 | .1057 |
| **Combat history (Yes)** | .181 | .108 | 1.67 | .0947 | .170 | .109 | 1.56 | .1189 |
| **Total encounters** | .004 | .020 | .22 | .8259 | .001 | .020 | .05 | .9624 |
| **Loneliness, baseline** | — | — | — | — | .011 | .017 | .62 | .5376 |
| **Model R^2^** |  |  |  | .037 |  |  |  | .038 |

Note. Positive β values indicate greater PTSD symptom improvement (i.e., larger reduction in PCL-5 scores). Step 1 model: N = 697, F(18, 678) = 1.47, p = .0958, R² = .037, adjusted R² = .012. Step 2 model: N = 692, F(19, 672) = 1.39, p = .1230, R² = .038, adjusted R² = .011. Reference groups are the omitted categories in the dummy-coded model. Encounter variables were winsorized.

**Supplemental Table W.**

*Hierarchical Regression Predicting* ***Dysphoric*** ***Arousal*** *Symptom Change, Based on Loneliness, Demographic Covariates, and* ***Medication Encounters*** *at Baseline*

| Predictor | Step 1 (covariates plus encounters) | | | | Step 2 (covariates plus encounters and loneliness) | | | |
| --- | --- | --- | --- | --- | --- | --- | --- | --- |
|  | β | SE | t | p | β | SE | t | p |
| **Intercept** | .274 | .408 | .67 | .5017 | .102 | .411 | .25 | .8043 |
| **Age group** |  |  |  |  |  |  |  |  |
| 26-35 | -.285 | .227 | -1.26 | .2093 | -.270 | .225 | -1.20 | .2315 |
| 36-45 | -.128 | .218 | -.58 | .5592 | -.129 | .217 | -.60 | .5520 |
| 46-55 | -.148 | .220 | -.67 | .5030 | -.141 | .219 | -.65 | .5188 |
| 56-65 | -.335 | .217 | -1.55 | .1226 | -.325 | .216 | -1.51 | .1321 |
| 66-75 | -.512 | .230 | -2.22 | .0266 | -.495 | .229 | -2.17 | .0307 |
| 76+ | -.528 | .258 | -2.05 | .0410 | -.502 | .256 | -1.96 | .0501 |
| **Race and ethnicity** |  |  |  |  |  |  |  |  |
| American Indian and Alaska Native | -.237 | .356 | -.67 | .5052 | -.255 | .353 | -.72 | .4697 |
| Asian American | -.149 | .247 | -.60 | .5455 | -.083 | .253 | -.33 | .7435 |
| Black/African American | .094 | .097 | .97 | .3312 | .070 | .096 | .73 | .4668 |
| Native Hawaiian and Pacific Islander | -.236 | .378 | -.62 | .5335 | -.271 | .376 | -.72 | .4704 |
| Hispanic/Latinx | -.268 | .147 | -1.82 | .0691 | -.258 | .146 | -1.76 | .0781 |
| More than one race | .248 | .220 | 1.13 | .2609 | .252 | .219 | 1.15 | .2496 |
| Unknown | -.201 | .132 | -1.53 | .1270 | -.237 | .132 | -1.80 | .0718 |
| **Marital status** | .103 | .079 | 1.31 | .1919 | .126 | .079 | 1.59 | .1127 |
| **Sex (Male)** | .092 | .094 | .97 | .3313 | .099 | .094 | 1.05 | .2938 |
| **Rurality (Urban)** | .164 | .091 | 1.81 | .0711 | .162 | .091 | 1.79 | .0741 |
| **Combat history (Yes)** | .124 | .081 | 1.53 | .1255 | .102 | .080 | 1.27 | .2046 |
| **Total encounters** | .008 | .015 | .52 | .6026 | -.002 | .015 | -.10 | .9176 |
| **Loneliness, baseline** | — | — | — | — | .030 | .013 | 2.34 | .0197 |
| **Model R^2^** |  |  |  | .045 |  |  |  | .053 |

Note. Positive β values indicate greater PTSD symptom improvement (i.e., larger reduction in PCL-5 scores). Step 1 model: N = 697, F(18, 678) = 1.76, p = .0265, R² = .045, adjusted R² = .019. Step 2 model: N = 692, F(19, 672) = 1.98, p = .0077, R² = .053, adjusted R² = .026. Reference groups are the omitted categories in the dummy-coded model. Encounter variables were winsorized.

**Supplemental Figure A**

*Mean PTSD-Related Anxious Arousal Symptoms by Loneliness Tertiles (Low, Medium, High)*

Anxious Arousal Score on PCL-5

| Timepoint | Low | Medium | High |
| --- | --- | --- | --- |
| Baseline | 2.3726 | 2.67824 | 2.94163 |
| Follow-Up | 1.85714 | 2.11468 | 2.34942 |

*Note*. Mean anxious arousal symptom scores on the *PTSD Checklist for the DSM-5* (PCL; Blevins et al., 2015) across low, medium, and high tertiles of loneliness are plotted against study timepoint (baseline and follow-up).

**Supplemental Figure B**

*Mean PTSD-Related Avoidance Symptoms by Loneliness Tertiles (Low, Medium, High)*

Avoidance Score on PCL-5

| Timepoint | Low | Medium | High |
| --- | --- | --- | --- |
| Baseline | 2.25721 | 2.58372 | 3.06008 |
| Follow-Up | 1.75 | 2.04817 | 2.49421 |

*Note*. Mean avoidance symptom scores on the *PTSD Checklist for the DSM-5* (PCL; Blevins et al., 2015) across low, medium, and high tertiles of loneliness are plotted against study timepoint (baseline and follow-up).

**Supplemental Figure C**

*Mean PTSD-Related Dysphoric Arousal Symptoms by Loneliness Tertiles (Low, Medium, High)*

Dysphoric Arousal Score on PCL-5

| Timepoint | Low | Medium | High |
| --- | --- | --- | --- |
| Baseline | 1.53125 | 1.93326 | 2.33333 |
| Follow-Up | 1.20714 | 1.55505 | 1.80695 |

*Note*. Mean dysphoric arousal symptom scores on the *PTSD Checklist for the DSM-*5 (PCL; Blevins et al., 2015) across low, medium, and high tertiles of loneliness are plotted against study timepoint (baseline and follow-up).

**Supplemental Figure D**

*Mean PTSD-Related Emotional NACM Symptoms by Loneliness Tertiles (Low, Medium, High)*

NACM Score on PCL-5

| Timepoint | Low | Medium | High |
| --- | --- | --- | --- |
| Baseline | 1.53033 | 2.07282 | 2.64147 |
| Follow-Up | 1.24966 | 1.63041 | 2.11418 |

*Note*. NACM = Negative alterations in cognitions and mood. Mean NACM symptom scores on the *PTSD Checklist for the DSM-5 (*PCL; Blevins et al., 2015) across low, medium, and high tertiles of loneliness are plotted against study timepoint (baseline and follow-up).

**Supplemental Figure E**

*Mean PTSD-Related Reexperiencing Symptoms by Loneliness Tertiles (Low, Medium, High)*

Reexperiencing Score on PCL-5

| Timepoint | Low | Medium | High |
| --- | --- | --- | --- |
| Baseline | 1.89543 | 2.2564 | 2.71027 |
| Follow-Up | 1.60286 | 1.83211 | 2.19691 |

*Note*. Mean re-experiencing symptom scores on the *PTSD Checklist for the DSM-5* (PCL; Blevins et al., 2015) across low, medium, and high tertiles of loneliness are plotted against study timepoint (baseline and follow-up).
